# Activation of IRE1, PERK and salt-inducible kinases leads to Sec body formation in *Drosophila* S2 cells

**DOI:** 10.1242/jcs.258685

**Published:** 2021-09-06

**Authors:** Chujun Zhang, Wessel van Leeuwen, Marloes Blotenburg, Angelica Aguilera-Gomez, Sem Brussee, Rianne Grond, Harm H. Kampinga, Catherine Rabouille

**Affiliations:** 1 Hubrecht Institute of the KNAW & UMC Utrecht, Utrecht, 3584 CT, The Netherlands; 2 Department of Biomedical Sciences in Cells and Systems, UMC Groningen, Groningen, 9713 AV,The Netherlands; 3 Section Cell Biology, Center for Molecular Medicine, University Medical Center Utrecht, Utrecht, 3584 CX, The Netherlands

**Keywords:** Phase separation, *Drosophila* S2 cells, Sec body, Amino acid starvation, Salt stress, Unfolded protein response

## Abstract

The phase separation of the non-membrane bound Sec bodies occurs in *Drosophila* S2 cells by coalescence of components of the endoplasmic reticulum (ER) exit sites under the stress of amino acid starvation. Here, we address which signaling pathways cause Sec body formation and find that two pathways are critical. The first is the activation of the salt-inducible kinases (SIKs; SIK2 and SIK3) by Na^+^ stress, which, when it is strong, is sufficient. The second is activation of IRE1 and PERK (also known as PEK in flies) downstream of ER stress induced by the absence of amino acids, which needs to be combined with moderate salt stress to induce Sec body formation. SIK, and IRE1 and PERK activation appear to potentiate each other through the stimulation of the unfolded protein response, a key parameter in Sec body formation. This work shows the role of SIKs in phase transition and re-enforces the role of IRE1 and PERK as a metabolic sensor for the level of circulating amino acids and salt.

This article has an associated First Person interview with the first author of the paper.

## Abstract (precis)

**Summary:** In S2 cells, the phase-separated Sec bodies form upon the combined activation of salt-inducible kinases, IRE1 and PERK.

## INTRODUCTION

Cell compartmentalization is not only mediated by membrane-bound organelles. It also relies on non-membrane bound biomolecular condensates (so-called membraneless organelles) that populate the nucleus and the cytoplasm.

The formation of membraneless organelles has been shown to occur through phase separation, which can be driven by stress (such as ER, oxidative, proteostatic or nutrient stress), resulting in the formation of stress assemblies (van Leeuwen and Rabouille, 2019). Those are mesoscale coalescence of specific and defined components that phase separate. For instance, nutrient stress leads to the formation of many biocondensates. Most of them are RNA based, such as stress granules and P-bodies (van Leeuwen and Rabouille, 2019), but some are not. This is the case for glucose-starved yeast where metabolic enzymes foci (Munder et al., 2016; Petrovska et al., 2014) and proteasome storage granules (Peters et al., 2013; van Leeuwen and Rabouille, 2019) form, as well as *Drosophila* S2 cells that form Sec bodies under conditions of amino acid starvation (Zacharogianni et al., 2014).

Sec bodies are related to the inhibition of protein secretion in the early secretory pathway. The early secretory pathway comprises the endoplasmic reticulum (ER), where newly synthesized proteins destined to the plasma membrane and the extracellular medium are synthesized. Proteins exit the ER at the ER exit sites (ERES) to reach the Golgi. The ERES are characterized by the concentration of COPI-coated vesicles whose formation requires six proteins, including Sec12 and Sar1, the inner coat proteins Sec23 and Sec24, and the outer coat proteins Sec13 and Sec31 (Gomez-Navarro and Miller, 2016). In addition, a larger hydrophilic protein called Sec16, has been identified as a key regulator of the ERES organization and COPII vesicle budding (Sprangers and Rabouille, 2015). Many additional lines of evidence support the role of Sec16 in optimizing COPII-coated vesicle formation and export from the ER (Farhan et al., 2010; Joo et al., 2016; Wilhelmi et al., 2016).

**Fig. 1.**
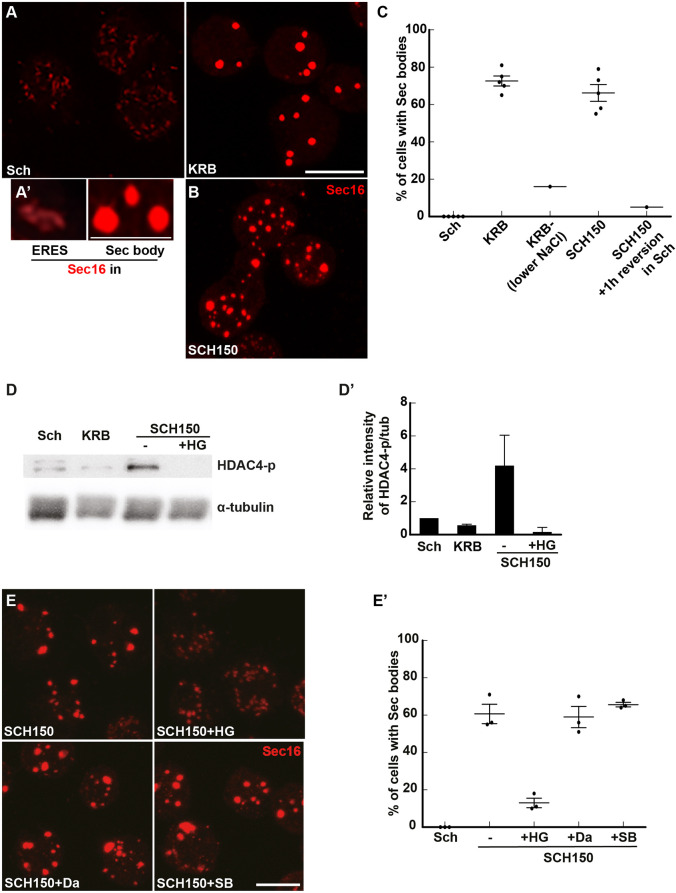
**Salt stress activates the SIKs, which are involved in Sec body formation.** (A,A′) Immunofluorescence (IF) visualization of endogenous Sec16 in S2 cells growing in Schneider's medium (Sch) and in cells incubated in KRB (A). Note the difference in the Sec16 pattern; Sec16 is at ER exit sites in growing cells and in Sec bodies in cells incubated in KRB. Upon KRB incubation, ERES remodel into larger structures, the Sec bodies, that are brighter than ERES. (B) IF visualization of Sec body formation (marked by Sec16) in cells incubated in Schneider's medium supplemented with 10 mM sodium bicarbonate and 150 mM of NaCl (SCH150) for 4 h at 26°C. (C) Quantification of Sec body formation (marked by Sec16) in cells incubated in Sch, KRB, KRB with lower NaCl (containing only 60 mM NaCl) and SCH150 for 4 h at 26°C as well as SCH150 and then in Sch for 1 h (reversion), showing that SCH150-induced Sec bodies are formed reversibly. (D,D′) Western blot of S2 cells protein extract after incubation in Schneider's medium (Sch), KRB and SCH150 with and without HG-9-91-01 (5 µM) for 4 h at 26°C blotted for HDAC4-p and α-tubulin. Quantification of the ratio HDAC4-p to α-tubulin (D′). (E,E′) IF Visualization (E) and quantification (E′) of Sec body formation (marked by Sec16) in cells incubated in SCH150 supplemented or not with the SIK inhibitor HG-9-91-01 (HG, 5 µM), the Src inhibitor dasatinib (Da, 20 µM) and the p38 MAPK inhibitor SB203580 (SB, 30 µM) for 4 h at 26°C. Scale bars: 10 µm. Errors bars: s.e.m.

Upon the stress of amino acid starvation in Krebs Ringer bicarbonate buffer (KRB), the ERES of *Drosophila* S2 cells are remodeled into large round non-membrane bound phase-separated Sec bodies. They are typically observed by immunofluorescence after staining of endogenous Sec16, Sec23 and expressed Sec24–GFP (Zacharogianni et al., 2014) (see Fig. 1A,A′). Importantly, Sec bodies are very quickly resolved upon stress relief (addition of growth medium). Finally, they appear to protect the components of the ERES from degradation (Zacharogianni et al., 2014) and they help cells to survive under conditions of amino acid shortage (Aguilera-Gomez et al., 2016; Zacharogianni et al., 2014).

Phase separation has been shown to be driven by specific components, the so-called drivers, either RNAs or proteins harboring structural features that become exposed or modified under certain conditions. In the case of Sec bodies, Sec24AB (Aguilera-Gomez et al., 2016; Zacharogianni et al., 2014) and Sec16 have been shown to drive Sec body coalescence (Aguilera-Gomez et al., 2016) in a manner that depends on a small stretch of 44 residues in Sec16 and on the mono-ADP-ribosylation enzyme by PARP16 (Aguilera-Gomez et al., 2016). This illustrates the critical role of post-translational modifications in phase separation (Bah and Forman-Kay, 2016; Owen and Shewmaker, 2019).

In parallel, changes in cytoplasmic biophysical properties have also been shown to be important in phase separation (Rabouille and Alberti, 2017), such as a drop of cytoplasmic pH within minutes, without post-translational modifications (Munder et al., 2016; Peters et al., 2013; van Leeuwen and Rabouille, 2019).

Here, we seek out to (1) identify the pathways elicited in S2 cells upon incubation in the starvation medium KRB that lead to Sec body formation, and (2) to assess whether changes in the cytoplasmic biophysical properties play a role in the phase transition leading to Sec body formation. We show that amino acid starvation in KRB stimulates ER stress and activation of two downstream kinases, IRE1 and PERK (also known as PEK in flies) leading to the stimulation of the unfolded protein response (UPR) (Boyce and Yuan, 2006; Walter and Ron, 2011). However, the sole activation of the IRE1 and PERK does not lead to Sec body formation. To form Sec bodies in KRB, IRE1 and PERK activation needs to be combined with a moderate salt stress. Accordingly, KRB incubation is faithfully mimicked by cell incubation with dithiothreitol (DTT) and addition of 100 mM NaCl. Interestingly, a high-salt stress addition of 150 mM NaCl, which activates the salt-inducible kinases (SIKs; SIK2 and SIK3), is sufficient to efficiently drive Sec body formation. Importantly, we found that a decrease in the cytoplasmic ATP concentration, a general RNA degradation and the stimulation of the UPR are factors strongly correlated to Sec body formation.

## RESULTS

### Salt stress is necessary and sufficient for Sec body formation

In an attempt to understand Sec body formation upon incubation in the starvation buffer KRB (Fig. 1A,A′), we noticed that the salt concentration of the Schneider's medium, in which S2 cells are grown, is much lower than that in mammalian tissue culture media [such as Dulbecco's modified Eagle's medium (DMEM)] and KRB, which is used as a starvation medium [for instance, 3-fold lower for Na^+^ (i.e. 51 mM in Schneider's medium and 138 mM in KRB); Table 1]. Accordingly, we found that lowering the concentration of NaCl in KRB (to 60 mM instead of the 120 mM in normal KRB (78 mM instead of 138 mM Na^+^, Table 1) resulted in a decrease in Sec body formation (Fig. 1C), showing that NaCl is necessary for their formation. Conversely, the addition of 150 mM NaCl together with 10 mM sodium bicarbonate (SCH150, resulting in 211 mM Na^+^; Table 1) to Schneider's medium led to a substantial formation of reversible Sec bodies, as efficiently as incubating the cells in KRB (Fig. 1A–C).

**Table 1.**
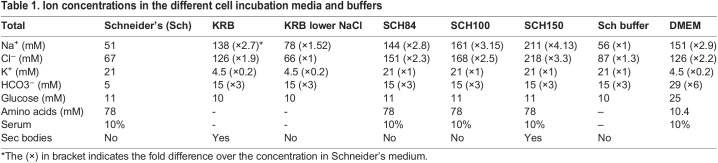
Ion concentrations in the different cell incubation media and buffers

Osmotic shock has been reported to trigger the formation of different stress assemblies, such as stress granules (Aulas et al., 2017; Bounedjah et al., 2012) and P-bodies (Jalihal et al., 2020; Kilchert et al., 2010). However, addition of 0.4–0.6 M sucrose does not elicit Sec body formation (Fig. S1C), even though the cell diameter significantly decreased by 22% (Fig. S1A,B). This shows that the shrinkage of the cell volume is not a factor leading to Sec body formation. Furthermore, neither the addition of 150 mM of KCl nor of sodium acetate instead of NaCl led to a substantial formation of Sec bodies (Fig. S1C). Taken together, these results suggest that increasing the Na^+^ concentration in Schneider's medium by 4-fold (referred to as salt stress), but not osmotic shock or K^+^ increase (Table 1), triggers a pathway that leads to Sec body formation.

To test this further, we aimed to increase cytoplasmic Na^+^ concentration and assess whether that was sufficient for Sec body formation by manipulating the abundant NaK ATPase present at the plasma membrane. NaK ATPase extrudes three Na^+^ ions against two K^+^ ions, and is the main pump that maintains the intracellular Na^+^ concentration low and compatible with cellular function. We found that the localization of NaK ATPase changes dramatically from intracellular puncta for cells in Schneider's medium to a strong plasma membrane localization in cells in SCH150 (Fig. S1D) together with a strong increase in its protein level (Fig. S1E), suggesting its involvement. Accordingly, incubation of cells with the NaK ATPase inhibitor ouabain in the growing medium SCH100 (161 mM Na^+^, Table 1) led to a robust Sec body formation (Fig. S1F,F′), presumably because the cytoplasmic Na^+^ concentration increases, as Na^+^ can no longer be extruded. This shows that increasing Na^+^ in the cytoplasm is sufficient to drive Sec body formation, perhaps by mobilizing the NaK ATPase.

We then asked which pathway is activated downstream of increased Na^+^. An increase in intracellular Na^+^ concentration is known to activate the SIKs, and in this regard, two SIKs (SIK2 and SIK3) are expressed in *Drosophila* (Teesalu et al., 2017; Wehr et al., 2013). To test this, we monitored the phosphorylation of a known SIK target, Histone Deacetylase 4 (HDAC4), which is phosphorylated upon SIK2 and SIK3 activation, and found that it was also strongly (4-fold) activated in SCH150. Importantly, this SCH150-induced phosphorylation is 97% inhibited by addition of a pan-SIK inhibitor HG-9-91-01 (HG) (Fig. 1D,D′).

We then used HG to test the role of SIK activation in Sec body formation. Addition of HG reduces Sec body formation in 85% of the cells incubated in SCH150 (Fig. 1E,E′). As HG can also inhibit p38 MAPKs and Src (Clark et al., 2012), we used specific inhibitors for these two groups of kinases and assessed their potential to inhibit Sec body formation with SCH150. Neither the p38 MAPK inhibitor SB203580 nor the Src inhibitor Dasatinib, inhibited Sec body formation (Fig. 1E,E′), showing that the effect of HG can be attributed to SIK inhibition.

The above results not only show that S2 cells are fully responsive to HG, but also that SIK activation by increased NaCl concentration in the medium (inducing Na^+^ stress) is a key pathway for Sec body formation.

### In addition to salt stress, amino acid starvation activates other pathways necessary for Sec body formation

**Fig. 2.**
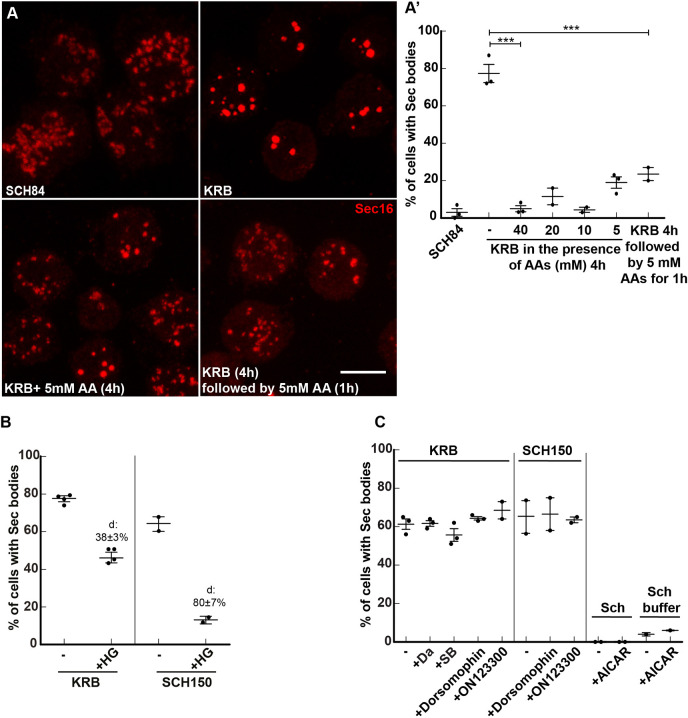
**Amino acid starvation enhances cell salt stress-induced Sec body formation.** (A,A′) Immunofluorescence (IF) visualization (A) and quantification (A′) of Sec body formation (marked by Sec16) in cells incubated in SCH84, KRB, KRB supplemented with 5–40 mM amino acids (AA) for 4 h at 26°C, as well as KRB for 4 h followed by addition of 5 mM amino acids for 1 h at 26°C. (B) Quantification of Sec body formation (marked by Sec16) in cells incubated in KRB and SCH150 with or without the SIK inhibitor HG-9-91-01(5 µM). (C) Quantification of Sec body formation (marked by Sec16) in cells incubated in KRB and SCH150 with or without the Src inhibitor dasatinib (Da, 20 µM), the p38 MAPK inhibitor SB203580 (SB, 30 µM), Dorsomorphin (1 µM) and ON123300 (10 µM) for 4 h at 26°C, as well in cells incubated in Schneider's medium (Sch) and Schneider's medium buffer supplemented with or without the AMPK activator AICAR (1 mM). ‘d’ indicates the mean±s.e.m. decrease in Sec body formation when compared to the absence of inhibitors. Scale bars: 10 µm. Errors bars: s.e.m.

The data above opens the possibility that the formation of Sec bodies in the starvation medium KRB is simply be due to an elevated salt concentration (Table 1), unrelated to the absence of amino acids. However, this is not the case. When the Na^+^ concentration in Schneider's medium and KRB are set to the same values (around 140 mM, KRB versus SCH84, Table 1), Sec bodies form very efficiently in KRB but not at all in SCH84 (Fig. 2A′). The major difference between these two media is that SCH84 contains 78 mM amino acids and serum, whereas KRB contains none. Since we have demonstrated previously that serum (that was dialyzed to remove free amino acids) does not play a role in Sec body formation (Aguilera-Gomez et al., 2016; Zacharogianni et al., 2014), this indicates that the presence of amino acids prevents Sec body formation even upon a moderate Na^+^ stress.

To demonstrate this further, cells were incubated with KRB supplemented or not with amino acids. Addition of 5–40 mM amino acids to KRB strongly prevented Sec body formation (Fig. 2A,A′), and led to the reversion of KRB-triggered Sec bodies back to ERES (Fig. 2A,A′), almost as efficiently as the reversion in Schneider's medium (Zacharogianni et al., 2014). This suggests that, for an equivalent salt concentration, the presence of amino acids indeed prevents Sec body formation. This shows that amino acid starvation is instrumental to Sec body formation by potentiating Na^+^ stress. Alternatively, the presence of salt might potentiate pathways induced by amino acid starvation leading to Sec body formation, perhaps through SIK activation. To test this, we incubated cells in KRB in the presence of the SIK inhibitor HG, and found that Sec body formation was inhibited by 38% (Fig. 2B), suggesting their involvement.

However, HDAC4 (the SIK target mentioned above) does not appear to be phosphorylated upon incubation in KRB (Fig. 1D,D′). As above, to rule out an unspecific inhibition of other kinases by HG, we tested p38 MAPK and Src inhibitors, but found that these inhibitors do not affect KRB-induced Sec body formation (Fig. 2C). Furthermore, as SIKs are member of the AMPK family, we tested whether known inhibitors of AMPK affect KRB-induced Sec body formation. However, neither Dorsomorphin (compound C) (Weiss et al., 2010), nor ON123300 (Zhang et al., 2014) affected this (Fig. 2C), as was also the case for SCH150, showing that salt stress is largely mediated by SIKs. Furthermore, addition of the AMPK agonist AICAR (Ducommun et al., 2014) to Schneider's medium, even in the absence of amino acids (Sch buffer), did not lead to Sec body formation. This suggests that no AMPK family members other than SIKs are required for Sec body formation in cells incubated in KRB. Either HDAC4 is not a SIK target in KRB, or the HADC4 protein is degraded in KRB, making the increased phosphorylation difficult to show experimentally.

As HG only inhibits KRB-triggered Sec body formation by 38%, not 80% as seen with SCH150 (Fig. 2B), these results show that the Sec body formation in KRB is the result of an interplay between Na^+^ stress (through increased NaCl concentration in the medium) and the absence of amino acids that triggers another stress. We therefore investigated which other pathways are triggered upon amino acid starvation in KRB.

### The sole inhibition of mTORC1 is neither sufficient nor necessary to trigger Sec body formation

Mechanistic Target of Rapamycin Complex 1 (mTORC1) is the major sensor of amino acid level in the circulating medium (Kim and Guan, 2019). When amino acids are absent or low, the complex is inhibited, resulting in the inhibition of many anabolic pathways, and the activation of the degradative pathway of autophagy. We have previously shown that in S2 cells incubated with KRB, protein synthesis is inhibited very quickly, and the mTORC1 target S6 Kinase is no longer phosphorylated (Fig. S2A), and the autophagic pathway is activated (Zacharogianni et al., 2014). We also showed that the sole inhibition of mTORC1 by Rapamycin (Zacharogianni et al., 2014) and Torin (Fig. S2B,B′) is not enough to trigger the formation of Sec bodies. Similarly, depleting Raptor, the main subunit of mTORC1, does not trigger Sec body formation in growing cells (Zacharogianni et al., 2014). Finally, overexpression of TCS1, an endogenous inhibitor of mTORC1 (Condon and Sabatini, 2019) also failed to trigger the formation of Sec bodies (Fig. S2C). Taken together, although amino acid starvation does inhibit mTORC1 in S2 cells, Sec body formation is not a consequence of its sole inhibition.

### KRB incubation leads to oxidative stress but oxidative stress alone is not enough to trigger Sec body formation

In order to determine which other pathways are stimulated by amino acid starvation in KRB, we performed bulk RNA sequencing and compared the results to those of cells grown in Schneider's medium. We detected 15,679 genes, including 1070 downregulated and 1635 upregulated (Fig. S3A*,* Table S1; see GEO: GSE143810). Interestingly, the strongest GO term associated with upregulated genes is oxidoreductase activity, including 20 genes expressing glutathione-S-transferase that are unregulated as well as a strong reduction in peroxiredoxin expression, suggesting that KRB could elicit oxidative stress (Fig. S3B), perhaps through reactive oxygen species (ROS) production.

To visualize the ROS production upon KRB incubation, we used DCF fluorescence measurements. KRB incubation elicits a specific, robust and steady increase of ROS production that is significantly inhibited by the ROS inhibitor N-acetyl-L-cysteine (NAC) (Fig. S3C). However, this does not prevent Sec body formation (Fig. S3D′). Furthermore, generating oxidative stress with arsenite (Fig. S3D,D′), ammonium persulfate (APS), or H_2_O_2_ (data not shown) in growing cells in Schneider's medium does not lead to Sec body formation. Taken together, ROS are produced during KRB incubation but this is not sufficient to trigger Sec body formation.

### KRB incubation stimulates IRE1 activation

Since Sec bodies are linked to the inhibition of the exit of newly synthesized proteins out of the ER, we investigated whether incubation in KRB stimulates ER stress, which is known to activate one or more of the three known downstream signaling pathways mediated by IRE1, PERK and ATF6 (Hetz, 2012; Korennykh et al., 2009; Moore and Hollien, 2012).

**Fig. 3.**
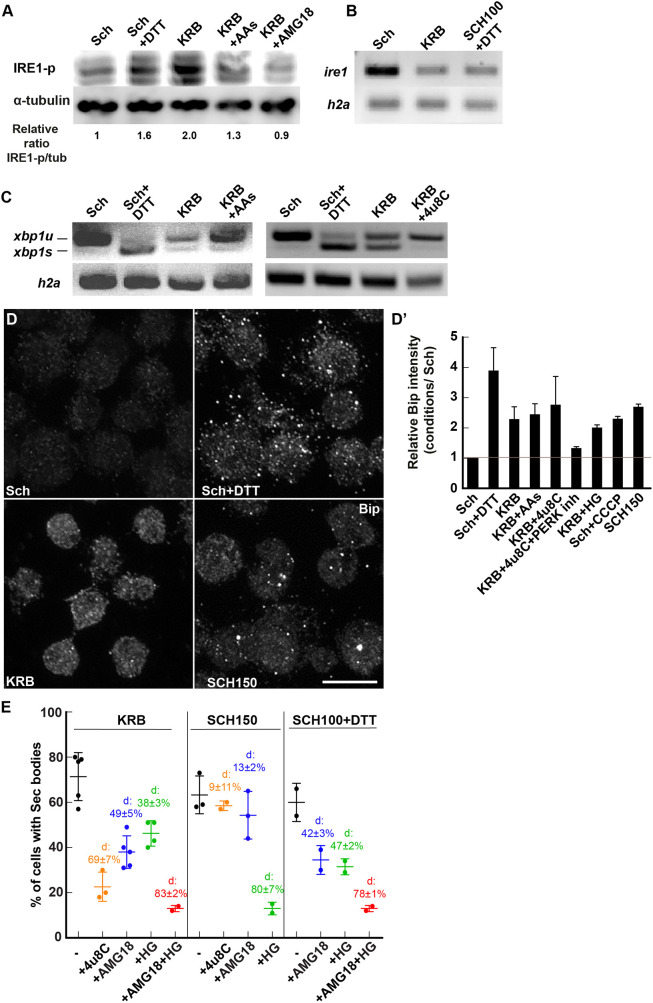
**KRB incubation activates IRE1.** (A) Western blot visualization of IRE1-p (using the anti IRE1-p antibody, Genentech) in cells in Schneider's medium (Sch), Sch+DTT (5 mM), KRB, KRB+amino acids (AAs) (5 mM) and KRB+AMG18 (10 µM) for 4 h after blotting. Note that KRB incubation elicits IRE1-p more strongly than Sch+DTT, and that addition of AAs to KRB partially reverses this phosphorylation. Addition of the IRE1 kinase attenuator AMG18 (10 µM) strongly inhibits IRE1-p formation. Quantification underneath is the ratio of IRE1-p (middle band) to α-tubulin for the blot shown. (B) Visualization of the PCR products of *ire1* and *h2a* mRNAs from cells incubated in Sch, KRB and SCH100+DTT for 4 h at 26°C. (C) Visualization of spliced (*xbp1s*) and unspliced (*xbp1u*) PCR products of *xbp1* upon conditions indicated on the panel. (D,D′) Immunofluorescence visualization (D) of the protein Bip in cells incubated in the conditions indicated on the panel. Quantification is in D′. Note that the UPR is stimulated in KRB and many other conditions. (E) Quantification of Sec body formation (marked by Sec16) in cells incubated in KRB, SCH150 or SCH100+DTT with or without 4u8C (30 µM), AMG18 (10 µM), HG (5 µM) or AMG18+HG. ‘d’ indicates the mean±s.e.m. decrease in Sec body formation when compared to the absence of inhibitors. *P*-value (SCH150 and SCH150+AMG18) is 0.104 and the *P*-value (KRB and KRB+HG) is 0.0019. The other differences are highly significant (<10^−4^). Errors bars: s.e.m. Scale bar: 10 µm.

IRE1 is an ER transmembrane protein that, when activated, dimerizes in the plane of the ER membrane, resulting in the autophosphorylation of its kinase domain (Walter and Ron, 2011). Cells incubated in the starvation medium KRB display a clear phosphorylated IRE1 (IRE1-p) signal when compared to cells in Schneider's medium, which is as strong as that seen upon addition of DTT, a known IRE1-activating agent (Fig. 3A). In the absence of antibodies to *Drosophila* IRE1, we tested whether the expression level of the kinase was modified by KRB treatment by monitoring the level of its mRNA by PCR, but this is not increased by this treatment (Fig. 3B). Importantly, we tested whether IRE1 activation was modulated by the presence of amino acids in the medium. We found that IRE1 is 34% less phosphorylated when KRB was replenished with amino acids than in KRB alone (Fig. 3A), partly sustaining the notion that amino acid starvation activates IRE1. Importantly, addition of the IRE1 kinase attenuator AMG18 (see below) completely prevented its phosphorylation (Fig. 3A).

The autophosphorylation of IRE1 in turn stimulates its nuclease activity, leading to the specific splicing of *xbp1* mRNA that is then translated into a potent transcription factor transcriptionally upregulating molecular machineries that resolve the overload of misfolded proteins in the ER (Walter and Ron, 2011), such as the UPR. IRE1 nuclease activity also triggers IRE1-dependent decay of mRNA (RIDD), a less-specific degradation of mRNAs that are largely associated to the ER (Hollien et al., 2009), which is also likely to relieve the ER burden of translocation and protein folding.

Both incubation in KRB and addition of DTT to Schneider's medium led to a clear spliced *xbp1* [*xbp1s*, representing 40% of the total in KRB, and 69% in DTT when compared to in Schneider's medium (Fig. 3C)]. In agreement with the decrease in IRE1-p, *xbp1* splicing in KRB was also 25% less pronounced in the presence of amino acids (Fig. 3C). This confirms that amino acid starvation leads, at least partly, to the activation of IRE1. Accordingly, inhibition of IRE1 nuclease activity by 4u8C totally inhibited *xbp1* splicing in KRB (Fig. 3C), suggesting that IRE1 is involved and that the drug works efficiently in S2 cells.

**Fig. 4.**
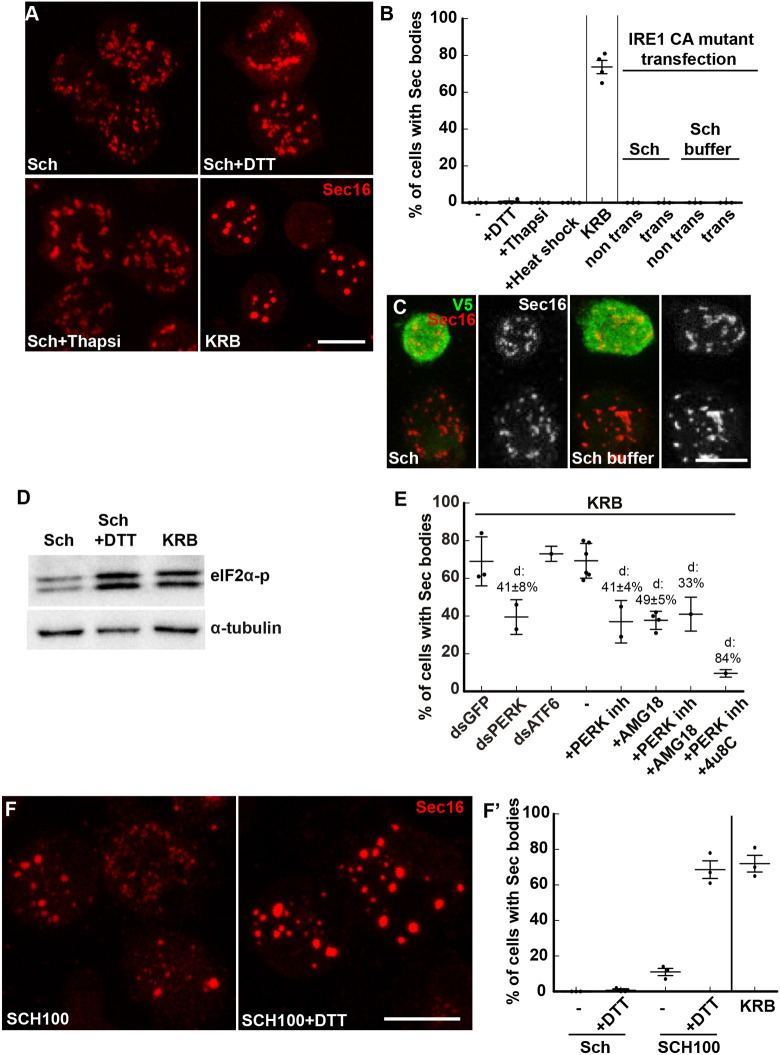
**KRB incubation is mimicked by a moderate salt stress combined with activation of IRE1 and PERK.** (A–C) Immunofluorescence (IF) micrographs of Sec16 in cells in Schneider's medium (Sch), KRB, Sch supplemented with DTT (5 mM) and thapsigargin (Thapsi, 2 µM) for 4 h at 26°C (A). Overexpression of the constitutively active (CA) IRE1 mutant tagged by V5 (in green) in cells incubated in Sch and Schneider's buffer (C). A quantification of the percentage of cells with Sec bodies is shown in B. Trans, transfected. (D) Western blot visualization of eIF2α-p in cells in Sch, Sch+DTT (5 mM) and KRB. (E) Quantification of Sec body formation (marked by Sec16) upon PERK depletion, PERK inhibition (5 µM), combined inhibition of PERK and IRE1 kinase (AMG18), and PERK and IRE1 nuclease (4u8C), as well as ATF6 depletion upon KRB incubation for 4 h at 26°C. ‘d’ indicates the mean±s.e.m. decrease in Sec body formation. (F,F′) Visualization (F) of Sec body formation (marked by Sec16) in cells incubated in SCH100 and SCH100+DTT (5 mM) for 4 h at 26°C. Quantification in F′. Errors bars: s.e.m. Scale bars: 10 µm.

To test whether activation of IRE1 triggers the UPR, we first monitored the Bip (also known as Hsc70-3 in flies) protein level, and found that this was increased 4-fold upon DTT addition (as expected) and 2.3-fold upon KRB, suggesting that the UPR is stimulated via IRE1 activation (Fig. 3D,D′). However, we found that 4u8C was only moderately powerful in suppressing Bip upregulation, suggesting that other pathways are likely to promote the UPR (such as that mediated by PERK, Fig. 4E, see below). Indeed only the combined inhibition of IRE1 and PERK suppressed Bip upregulation (Fig. 3D′). Surprisingly, we also found that incubating S2 cells in high salt (SCH150) also triggered an increase in Bip protein level, suggesting a yet-to-be-elucidated interplay between salt stress and the UPR stimulation (Fig. 3D′). This appears to be independent of SIK activation, as HG addition did not modify the observed Bip upregulation (Fig. 3D′).

To further test the stimulation of the UPR in KRB, we also monitored the level of UPR RNA targets*,* such as *bip* RNA, *xbp1* itself and *gadd45* (Fig. 3C, Fig. S4A,D). All were elevated upon addition of DTT, as expected (Fig. S4A,D*),* but not in KRB. In fact, except for *gadd45*, which did not change, the level of both *bip* and *xbp1* mRNAs were only 70% of the level in Schneider's medium, suggesting that these RNAs are partially degraded (Fig. S4A,D), making it difficult to precisely evaluate the stimulation of the UPR.

To test RIDD, we monitored the mRNA level of three RIDD targets, *indy*, *PIG-Wa* and *sparc* (Gaddam et al., 2012; Hollien and Weissman, 2006). We found that both *indy* and *PIG-Wa* (but not *sparc*) expression was lower in cells incubated in KRB when compared to cells in Schneider's medium (Fig. S4B,D). These results suggest that KRB incubation leads to IRE1 activation in turn activating the UPR, but also to RNA degradation, perhaps via RIDD.

If RIDD is the mechanism underlying the observed decreased level of these RNAs, it should be inhibited in the presence of 4u8C. We therefore monitored *bip* and *indy* RNA levels under this condition, but this treatment did not strongly rescue the *bip* and *indy* level (Fig. S4C,D) suggesting that the overall RNA degradation observed in KRB is likely not to occur via RIDD. In parallel, we tested the effect of the amino acid starvation on the degradation of these mRNAs (UPR and RIDD targets), and found that they all are rescued by addition of 5 or 40 mM amino acids (Fig. S4A,B,D). These results indicate that RNA degradation is downstream of the amino acid starvation but does not occur not via RIDD. Altogether, we conclude that KRB activates IRE1, the UPR and a non RIDD general pathway of RNA degradation that is strongly modulated by the absence of amino acids.

### IRE1 activation is necessary for Sec body formation but not sufficient

We then asked whether IRE1 activation is necessary for Sec body formation. To test this, we used to the IRE1 kinase activity attenuator AMG18 and 4u8C to monitor KRB-induced Sec body formation. Both inhibitor treatments substantially inhibited Sec body formation (49% for AMG18 and 69% for 4u8C) (Fig. 3E), indicating that IRE1 activity is necessary for Sec body formation. Of note, AMG18 is fully functional on *Drosophila* IRE1. Indeed, the kinase site of *Drosophila* IRE1 is very similar to the human enzyme (Fig. S4E). Furthermore, using AMG18 at either 10, 20 or 50µM during the KRB incubation inhibits Sec body formation to the same extent (Fig. S4F). Finally, the drug is 84% effective at blocking IRE1-p formation induced by thapsigargin (Fig. S4G). These results show that AMG18 is fully active in S2 cells.

The above results show that IRE1 activation is necessary for Sec body formation. However, it is not sufficient. First, activating IRE1 (through incubation with DTT) does not lead to Sec body formation (Fig. 4A,B). This is further supported by the finding that expression of a constitutive active form of IRE1 in which the three serine residues of the catalytic site have been mutated to aspartic acid (S703D, S705D and S708D) (Chang et al., 2018b; Yan et al., 2019) also does not lead to Sec body formation (Fig. 4B,C). This indicates that in addition to IRE1 activation, other pathways are stimulated.

As mentioned above, ER stress can also activate PERK (Hetz, 2012). In this regard, we found that eIF2α, an important PERK target that regulates translation, is strongly phosphorylated in cells in KRB, as much with addition of DTT to Schneider's medium (Fig. 4D). We then tested whether PERK activity was required for Sec body formation. Both PERK depletion by RNAi and PERK pharmacological inhibition by GSK2606414 led to a 50% reduction of Sec body formation upon KRB incubation (Fig. 4E). On the other hand, ATF6, the third pathway downstream of ER stress is not involved, as of ATF6 depletion by RNAi did not modify the degree of KRB-induced Sec body formation (Fig. 4E). This suggests that, parallel to IRE1 activation, PERK activity (but not ATF6) is also necessary for Sec body formation. This is also supported by the fact that combined inhibition of IRE1 and PERK in KRB leads to an 84% inhibition of Sec body formation (Fig. 4E). This is in line with the combined inhibition of PERK and IRE1 inhibiting the stimulation of the UPR (Fig. 3D′).

### A combination of moderate salt stress with IRE1 and PERK activation leads to Sec body formation

Although addition of DTT to Schneider's medium activates both IRE1 and PERK, it still does not lead to Sec body formation (Fig. 4A,D). Given the effect of the SIK inhibitor HG on KRB-stimulated Sec body formation (suggesting of SIKs activation) (Fig. 2B), we tested whether Sec body formation in KRB is recapitulated by activating IRE1 and PERK activation through DTT (5 mM) together with applying a moderate salt stress [SCH100 (161 mM Na^+^), equivalent to KRB (138Mm Na^+^); Table 1]. Strikingly, we found that SCH100 plus DTT (Fig. 4F,F′) is as efficient as KRB in inducing the formation of largely reversible Sec bodies. To assess further the equivalence between SCH100 plus DTT and KRB, we again used AMG18 and 4u8C (Fig. 3E), combined with the SIK inhibitor HG. AMG18 and 4u8C incubation resulted in a similar decrease in the Sec body response in both KRB and SCH100 plus DTT (∼45%). When IRE1 and SIK were inhibited together, the Sec body formation was also similarly strongly inhibited (Fig. 3E).

Taken together, Sec body formation in KRB is largely recapitulated by a moderate salt stress activating SIKs combined with ER stress triggered by amino acid starvation, which activates IRE1 and PERK (SCH100 plus DTT).

### The cytoplasm acidifies upon KRB incubation

As mentioned in the introduction, signaling pathways and/or change in the biophysical properties of the cytoplasm (Rabouille and Alberti, 2017; van Leeuwen and Rabouille, 2019) are critical for phase separation leading to the formation of stress assemblies. We therefore questioned how the biophysical cytoplasmic features are affected by the activation of these two pathways.

In glucose-starved yeast, the phase separation of proteasome subunits into proteasome storage granules and the formation of metabolic enzymes foci have been shown to occur through a drop in the cytoplasmic pH in a necessary and sufficient manner (Munder et al., 2016; Peters et al., 2013; van Leeuwen and Rabouille, 2019). We hence asked whether the cytoplasmic pH of the S2 cell cytoplasm also changed upon incubation in KRB and whether this is relevant to Sec body formation.

To do this, we used a cell-based assay to estimate the ratio of intensity of pHluorin versus mCherry in cells transfected with a pHluorin–mCherry cytoplasmic probe (Brett et al., 2005; Miesenböck et al., 1998). pHluorin is a pH-sensitive GFP mutant that changes its fluorescent spectrum at certain pHs. Upon KRB incubation, we observed that the cytoplasmic pH decreased, illustrated by a decrease in the ratio of pHluorin to mCherry when compared to cells grown in Schneider's medium where it remains largely constant (Fig. S5A,A′). We estimate that the cytoplasm of growing cells is at pH 6.8, whereas the pH of the cytoplasm of KRB-incubated cells is 6 (Fig. S5A,A′), suggesting a drop of nearly 1 pH unit. Interestingly, this result suggests that, similar to what occurs in yeast, nutrient starvation leads to a reduction of the cytoplasmic pH pointing to a conserved mechanism (Rabouille and Alberti, 2017; van Leeuwen and Rabouille, 2019). However, we found that arsenite treatment of S2 cells also leads to a similar pH drop. Given that this treatment does not lead to Sec body formation and this indicates that the decrease in pH is likely not to be a prime driver for Sec body formation.

### Sec body formation is largely associated to a drop in the cytoplasmic ATP concentration

**Fig. 5.**
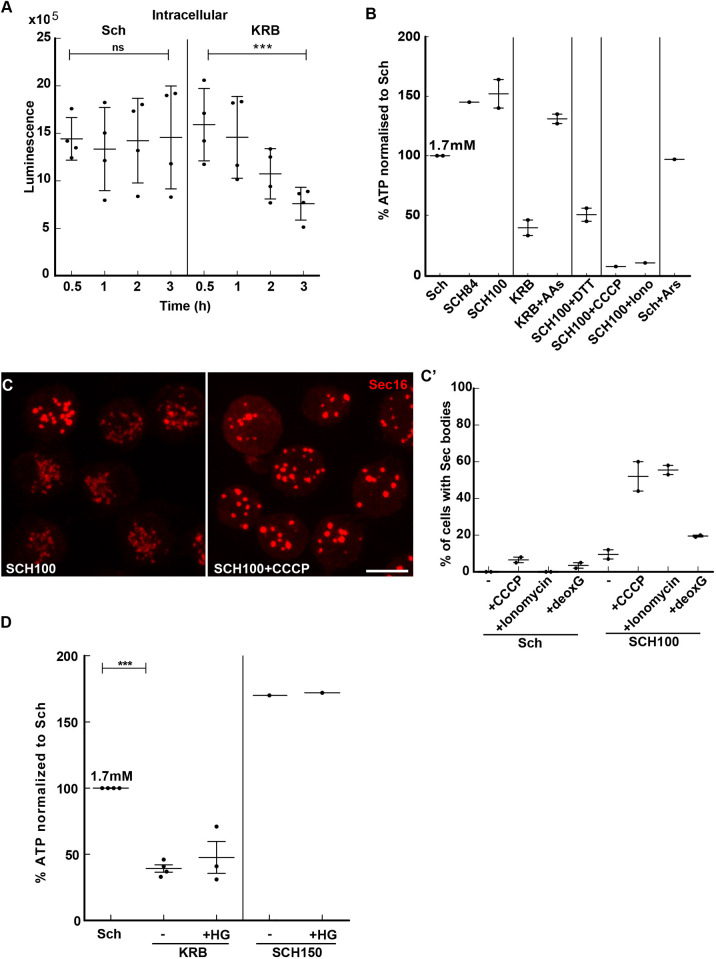
**The decrease of the intracellular ATP level is one driving factor for Sec body formation.** (A) Luminescence intensity measuring the intracellular ATP concentration of S2 cells during incubation in Schneider's medium (Sch) and KRB. Note that the ATP concentration decreases by 48% after 4 h incubation in KRB. ns, not significant; ***P<0.001. (B) Quantification of the change in the intracellular ATP concentration (percentage when compared to Sch, which is set to 100%) after 4 h incubation in the different conditions presented on the panel (see Table 1 and Table S2). AA, amino acids; Iono, ionomycin; Ars, arenite. (C,C′) Visualization of Sec16 (C) and quantification (C′) of Sec body formation (marked by Sec16) in cells supplemented by CCCP (25 µM), ionomycin (2.8 µM) and 2-deoxyglucose (deoxG; 20 mM) for 4 h at 26°C. (D) Quantification of the change in the intracellular ATP concentration (percentage when compared to Sch, which is set to 100%) upon SIK inhibition with HG-9-91-01 (HG, 5 µM) in KRB and SCH150. Scale bar: 10 µm. Errors bars: s.e.m.

In glucose-starved yeast, the pH drop has been shown to be the consequence of a decrease in the intracellular ATP concentration (due to a reduction of glycolysis) (Munder et al., 2016). Using a luciferase-based assay, we therefore measured the intracellular ATP concentration in S2 cells in KRB. The intracellular concentration steadily and specifically decreases after 1 h incubation in KRB-incubated cells (Fig. 5A). We estimate the intracellular ATP concentration to be 1.7 mM (Fig. 5B) in growing cells, a concentration that decreases by 50% after 3 h incubation in KRB (Fig. 5A,B). These results show that the sharp decrease in intracellular ATP concentration correlates with Sec body formation.

To investigate further whether the decrease in cytoplasmic ATP could be a driving factor in Sec body formation, we assessed the ATP level in experimental conditions that induce Sec body formation (Fig. 5B). In particular, incubation in SCH100 plus DTT induces a severe drop in the intracellular ATP. Furthermore, incubation with ouabain leads to an ATP decrease (and Sec body formation; Fig. S1F). Conversely, in cells incubated in KRB supplemented with 5 mM amino acids, where Sec body formation is largely inhibited, the intracellular ATP concentration is not decreased (Fig. 5B). Similarly, arsenite (which also does not lead to Sec body formation) also does not cause a drop in ATP. Overall, the conditions where Sec bodies form appear to be those where the ATP is the lowest.

To test whether a drop in intracellular ATP drives Sec body formation, we used two approaches. We first inhibited the ATP production of growing cells by blocking the mitochondrial OXPHOS with CCCP and ionomycin (Fig. 5C,C′), as well as by blocking glycolysis with 2-deoxyglucose. Strikingly, cells incubated in SCH100 supplemented by either CCCP or ionomycin had a robust Sec body formation, supporting the idea that a drop in intracellular ATP induces the formation of Sec bodies (Fig. 5C,C′). Incubation with 2-deoxyglucose also induced a consistent but small degree of Sec body formation (Fig. 5C′), suggesting that S2 cells rely more on mitochondrial respiration than on glycolysis.

**Fig. 6.**
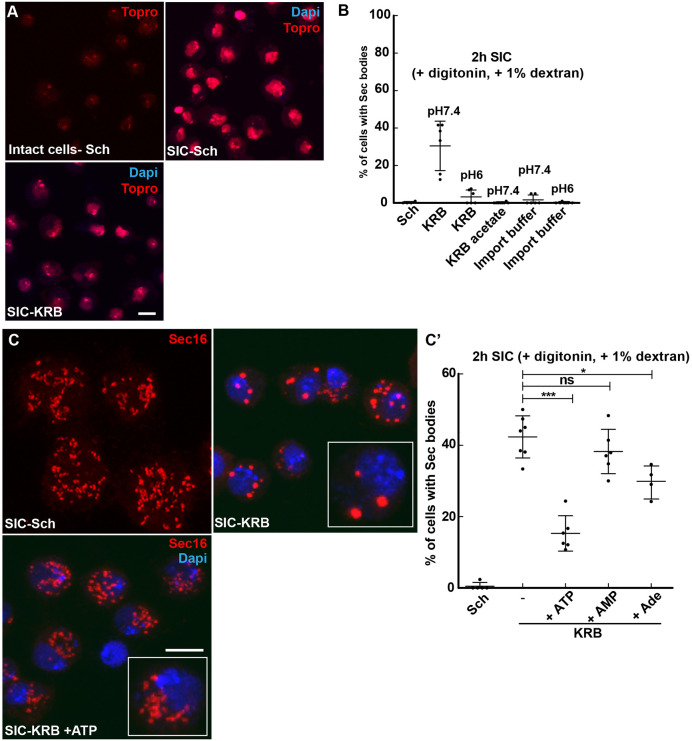
**Addition of ATP to semi-intact cells prevents Sec body formation.** (A) Visualization of S2 cells permeabilization using the non-membrane permeant TO-PRO-3 in intact cells in Schneider's medium (Sch) and in semi-intact cells (SICs;+10 µg/ml digitonin,+1% dextran) incubated in Sch and KRB for 2 h at 26°C. Note that TO-PRO-3 stains the nucleus only in the SIC system. (B) Effect of buffer composition in the formation of Sec bodies in SICs for 2 h at 26°C. Decreasing the pH of KRB from 7.4 to 6 decreases the efficiency of Sec body formation. Replacing Cl^−^ by acetate in the KRB and replacing KRB by the import buffer (20 mM HEPES, 110 mM KAc, 2 mM MgAc and 0.5 mM EGTA) abolishes Sec body formation. (C,C′) Immunofluorescence visualization of Sec16 (red, C) and quantification of Sec body formation (C′) (marked by Sec16) in the SIC system for cells incubated in Sch, in KRB and in KRB supplemented with 0.5 mM ATP, 0.5 mM AMP and 0.5 mM Adenosine. Cells in the white box are magnified 2.5 times. Scale bars: 10 µm. Errors bars: s.d. ns, not significant; *P<0.05, ***P<0.001.

Second, we developed a semi-intact cell (SIC) system using digitonin to gently permeabilize the S2 cell plasma membrane (Fig. 6A). The specific incubation of the permeabilized cells with KRB at pH 7.4 for 2 h led to Sec body formation in 38% of the cells (Fig. 6B). We confirmed that the pH of the buffer and the presence of NaCl is critical, as KRB at pH 6 and replacing Cl^−^ in the KRB with acetate did not lead to Sec body formation (Fig. 6B). To test the role of a low concentration of ATP in Sec body formation, KRB was supplemented with 0.5 mM ATP and this led to a strong inhibition of Sec body formation (Fig. 6C,C′). This is specific for ATP, as addition of AMP or adenosine did not lead to this strong inhibition (Fig. 6C,C′). These two results indicate that the intracellular ATP concentration modulates the phase separation of Sec bodies.

We then assessed whether the ATP drop is upstream or downstream of ER stress (activation of both IRE1 and PERK leading to UPR). To test this, we monitored the Bip protein level in CCCP-treated cells (Fig. 3D′) and found that it was increased, as in KRB. This suggests that lowering ATP cytoplasmic concentration appears to be upstream of ER stress and UPR activation. Taken together, the lowering in the cytoplasmic ATP concentration appears to be a key factor driving Sec body formation.

However, in conditions of high-salt stress through SCH150, the cytoplasmic concentration of cytoplasmic ATP does not change. Furthermore, addition of the SIK inhibitor has also no effect on the ATP change (Fig. 5D). This suggests that SIK activity is not instrumental to this ATP drop, and that Sec bodies can form even when the concentration of the ATP is not lowered.

### RNA degradation

The above results show the decrease in the ATP cytoplasmic concentration does not appear to be a common factor underlying Sec body formation in both KRB and SCH150. In search for a communality, we further investigated the overall decrease in RNA levels that we observed in both cells incubated in KRB and SCH150 (Fig. S5C). This decrease was also more specifically measured for *bip*, *xbp1*, *indy* and *PIG-Wa* (Fig. S4C). We therefore explored the possibility that Sec body formation is linked to RNA degradation. We first examined whether Sec bodies form to protect a set of RNAs from degradation, similar to what is thought to be the case for stress granules (Leung et al., 2006). We first monitored whether Sec bodies contain full-length RNAs by performing single-molecule fluorescence *in situ* hybridization (smFISH) using an oligo(dT) probe. As expected, stress granules (which also form upon incubation in KRB, marked here by the RNA-binding protein FMR1; Aguilera-Gomez et al., 2016), strongly colocalize with polyA-tailed RNAs, but Sec bodies are negative (Fig. S5D). This indicates that Sec bodies do not contain polyA-tailed RNAs, at least not in sufficient amounts to be detected by this method.

We then explored whether Sec bodies template around degraded RNAs. To test this, we used the SIC system of cells incubated in Schneider's medium where Sec bodies do not form (Fig. 6C), to which was added RNase 1 to degrade RNAs to nucleotides. However, this did not lead to Sec body formation (not shown). Finally, we added the total pool of RNAs from cells incubated in KRB to the SIC system in Schneider's medium. However, this also did not lead to Sec body formation (not shown). Consequently, in our hand, RNA degradation is not functionally linked to Sec body formation.

In conclusion, we propose that the UPR activation both by amino acid starvation in KRB and salt stress in SCH150 is more likely to be a key factor in Sec body formation.

## DISCUSSION

Here, we show that the Sec bodies that form in *Drosophila* S2 cells incubated in KRB are fully recapitulated by activation of SIKs, IRE1 and PERK (through SCH100 plus DTT), leading to the activation of a downstream UPR. Strikingly, the strong activation of SIKs in (SCH150) also induces the UPR and leads to Sec body formation. The resulting structures in each condition appear to be similar in size and number, and their formation is reversible. Whether their content is strictly similar has not been addressed here.

**Fig. 7.**
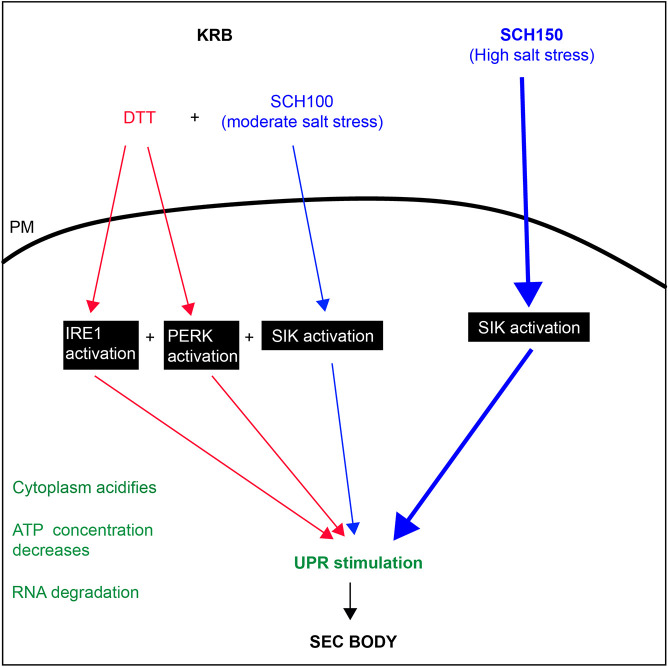
**Activation of SIK, IRE1 and PERK and SIK to form Sec bodies.** Blue pathway, Sec bodies can form upon high-salt stress (SCH150) through SIK activation, which, when strong is sufficient. A moderate salt stress also activates SIKs, but it is not enough to stimulate Sec body formation. Red pathway: amino acid starvation in KRB leads to IRE1, PERK and SIK activation leading to Sec body formation (mimicked by SCH100+DTT). IRE1 and PERK activation are necessary but not sufficient. In green are features appearing upon KRB incubation, such as cytoplasm acidification, a decrease in the cytoplasmic ATP concentration and the stimulation of the UPR that we propose to be a key factor in Sec body formation.

Taken together, our results show that Sec body formation requires the stimulation of two main signaling pathways (Fig. 7). The first is the salt stress pathway (addition of 150 mM NaCl), which activates the SIKs in a necessary and sufficient manner. It also does not lead to a change in the cytoplasmic ATP concentration. It does induce RNA degradation and it stimulates the UPR in an unexpected manner, given that PERK and IRE1 inhibitors do not alter SCH150 driven Sec body formation (Fig. 7).

The second pathway is the activation of IRE1 and PERK (but not ATF6), downstream of ER stress, which is partly induced by the absence of amino acids in KRB. Activation of either IRE1 or PERK is necessary but not sufficient. To form Sec bodies, this activation needs to be combined with a moderate salt stress. We propose that IRE1 and PERK activation combined with SIK activation occur in KRB, which is recapitulated by SCH100 plus DTT. This is associated with a decrease in the cytoplasmic concentration of ATP, with RNA degradation and with a stimulation of the UPR.

Interestingly, both strong salt stress (SCH150) and KRB lead to the activation of the UPR (measured by the increase in Bip protein level), leading to the possibility that SIKs, IRE1 and PERK interact with and/or activate, each other. Either IRE1 and/or PERK activate the SIKs, or SIK activation activates IRE1 and/or PERK. This still needs to be refined.

### The prominent role of salt stress and SIKs in remodeling the cytoplasm

Strong salt stress is induced by a 4-fold increase of Na^+^ in the medium combined with bicarbonate. This triggers an increase of Na^+^ in the cytoplasm that activates one or more SIK (as shown by the phosphorylation of the SIK target HDAC4). Accordingly, SIK inhibition decreases Sec body formation.

Keeping intracellular Na^+^ as near as possible to physiological concentrations (5 mM) is critical for cellular life, and the cell spends 40% of its available ATP to extrude Na^+^ against K^+^ with the NaK ATPase (Jaitovich and Bertorello, 2010). It is therefore not surprising that Na^+^ stress would elicit a cytoprotective response, such as prominent as Sec body formation (and stress granule formation in mammalian cells; Yan et al., 2014). This will need to be further elucidated, as many organisms and tissues are subjected to increased circulating Na^+^. We show, however, that it is not equivalent to an osmotic shock and that this addition of salt does not lead to a decrease in a cell volume. In contrast to P-bodies in yeast (Jalihal et al., 2020; Kilchert et al., 2010), osmotic stress does not induce Sec bodies. Interestingly, Na^+^/salt stress has recently been shown to induce the biogenesis of the lysosomal pathway (i.e. more endo/lysosomes as well as an increase in its activity) via TFEB and TOR (Lopez-Hernandez et al., 2020).

Increased Na^+^ activates the intracellular Na^+^-sensor network revolving around the SIKs (Jaitovich and Bertorello, 2010). The SIKs belong to the family of AMPKs, and have been shown to be part of a nutrient-sensing mechanism so far revolving around glucose (Wein et al., 2018) and unbalance of the ATP-to-ADP ratio. In mammals, there are three genes encoding SIK (SIK1–SIK3) but only 2 in *Drosophila*. *Drosophila* SIK2 is the ortholog of human SIK1 and SIK2, and has been shown to have a link to the fly Hippo pathway (Wehr et al., 2013), possibly linking nutrient to growth. *Drosophila* SIK3 is required for glucose sensing in the fly (Teesalu et al., 2017). Which SIK is involved in Sec body formation has not been clarified, as overexpression of each SIK individually has not proven enough to trigger Sec body formation, even when combined to some excess salt (SCH84 or SCH100). However, at least two SIKs appear to change their intracellular localization in KRB, that is, SIK2 and the long SIK3 isoform, which appear to cluster near the plasma membrane and localize to the nuclear envelope (data not shown)*.* The role of SIKs in the formation of stress assemblies (here the Sec bodies) appears important and novel, and needs to be investigated further. Other members of the AMPK family do not appear to be involved and changing the ratio ADP-to-ATP did not alter Sec body formation in our SIC system (not shown).

### IRE1 and PERK activation combined to SIK activation

Although a high salt stress is sufficient to trigger Sec body formation, the Sec body formation observed during incubation in the amino acid starvation buffer KRB elicits another pathway, the ER stress pathway, leading to the activation of both IRE1 and PERK. Indeed, KRB-induced Sec body formation is entirely mimicked by a moderate salt stress (SCH100, Table 1) combined with activation of IRE1 and PERK induced by DTT (SCH100 plus DTT).

### UPR stimulation

Surprisingly, we find that salt stress as well as KRB induces the UPR, which we find is a common downstream event in all conditions inducing Sec body formation. How salt stress activates the UPR and the exact role of IRE1 and PERK is still not fully understood. It does not appear to be via SIKs, as HG does not modulate the UPR, yet strongly reduces Sec body formation. Conversely, IRE1 and PERK inhibitors do not influence SCH150-induced Sec body formation, so the exact link between IRE1, PERK, SIKs and the UPR remains to be further investigated.

How UPR stimulation induces Sec body formation is not completely understood. It could occur through the clustering of IRE1 and PERK, two membrane kinases, and them forming a template in the plane of the ER membrane. In this regard, UPR stimulation has been linked to the MARylation enzyme *Drosophila* PARP16 (Jwa and Chang, 2012), which is also a transmembrane protein of the ER that undergoes a remodeling in KRB (Aguilera-Gomez et al., 2016). What other roles are played by events downstream of the UPR, for example, Bip upregulation itself, and cytoplasmic changes, remains to be elucidated in detail. One interesting aspect is whether the activation of UPR might affect biophysical properties, membrane association dynamics, and conformation and post-translational modifications of Sec16 and COPII subunits, which would lead to their enhanced phase separation properties under stress.

### Lowering the cytoplasmic ATP concentration is one parameter that induces Sec body formation

The lowering of the cytoplasmic ATP concentration occurs in KRB and SCH100 plus DTT. Forcing this lowering or preventing it has a strong incidence on Sec body formation. This finding is consistent with the hydrotropic property of ATP, that is, it being able to prevent phase separation in the cytoplasm. At least *in vitro*, addition of 8 mM ATP dissolves or prevents the phase separation of purified FUS and TAF15 (Patel et al., 2017). This is a concentration matching physiological range of ATP level in mammalian cells (1–10 mM) (Traut, 1994) and that is also compatible with the S2 cell ATP concentration (1.7 mM).

One possibility to explain the decrease of ATP concentration is the activation of kinases (among them, IRE1, PERK and SIK) that would consume the ATP pool. However, we propose that the decrease in the intracellular ATP concentration is upstream of the kinase activation. Indeed, incubating cells in KRB induces a ROS shock (as we showed experimentally), which could damage mitochondrial respiration, resulting, in turn, in ER stress, and UPR activation. Taken together, although we unravel a strong causality between low ATP and Sec body formation, the noticeable exception of such formation in SCH150 suggests other possibilities.

In conclusion, this work illustrates the complexity of amino acid starvation, the number of pathways it activates and how they interact with each other, as well as the different cellular cytoplasmic biophysical parameters it affects. We propose that the formation of Sec bodies depends on the activation of signaling pathways leading to activation of SIKs, IRE1 and PERK, altogether leading to the activation of the UPR, one of the common features of all pathways leading to Sec body formation.

## MATERIALS AND METHODS

### Cell culture, KRB incubation, drug treatments and depletions by RNAi

*Drosophila* S2 cells (R69007, Thermo Fisher Scientific) were cultured in Schneider's medium (Sch, S0146; Sigma) supplemented with 10% insect-tested fetal bovine serum (F4135; Sigma) at 26°C. S2 cells (between passages 5 and 18) were pelleted at 200 ***g*** in a microfuge for 3 min, washed once in fresh Schneider's medium, and diluted to 10^6^/ml. 1 ml of cell suspension were plated per well in a 12-well plate containing coverslips. Cells were allowed to attach for 1.5 h before starting the treatment.

Amino acid starvation was performed in Krebs Ringers bicarbonate buffer (KRB) comprising 0.7 mM Na_2_HPO_^4^_, 1.5 mM NaH_^2^_PO_^4^_, 15 mM NaHCO_^3^_^ ^(sodium bicarbonate, BIC), 120.7 mM NaCl, 4.53 mM KCl, 0.5 mM MgCl and 10 mM flucose at pH 7.4 as reported in Zacharogianni et al. (2014) (Table 1). SCH84, SCH100 and SCH150 correspond to Sch supplemented with 84, 100 and 150 mM NaCl. KRB21 corresponds to KRB supplemented to 21 mM KCl.

Wild-type *Drosophila* S2 cells were depleted by dsRNA, as previously described (Kondylis and Rabouille, 2003). Cells were analyzed after incubation with dsRNAs for 5 days, typically leading to depletion in more than 90% of the cells. Primers for depletion used were: dsPERK forward, 5′-TAATACGACTCACTATAGGGAGCTGGAGCTGGCTGTTTT-3′; dsPERK reverse, 5′- TAATACGACTCACTATAGGGTACTGGCGGATATCGGCTTC-3′, dsATF6 forward, 5′- TAATACGACTCACTATAGGGAGCGGCATGTCATAGCTGTA-3′, dsATF6 reverse, 5′- TAATACGACTCACTATAGGGTTGACGAGAAATGCAATCCA-3′.

### Cell treatments

Drugs were used at the concentrations mentioned in Table S2, including the IRE1 kinase inhibitor amgen small molecule 18 (AMG18) (Harrington et al., 2015). Drug treatment was performed on the plated cells at 26°C for 4 h incubated either in Schneider's medium or in KRB or any of the modified versions of these two media (Table 1). Heat shock was performed on 2×10^6^ S2 cells plated on coverslip in 3 cm dish at 38°C in an incubator for 1 h.

### Antibodies

For immunofluorescence, we used the rabbit polyclonal anti-Sec16 (1:800; Ivan et al., 2008) to detect Sec16, mouse monoclonal antibody a5 (1:20, deposited by Fambrough, D.M., DSHB) to detect NaK ATPase, mouse monoclonal anti-FMR1 (1:20, deposited by Siomi, H. DSHB) and rabbit polyclonal anti-GRP78 (Bip) (1:100, SPC-180, StressMarq) (Coelho et al., 2013; Sunderhaus et al., 2019). Donkey anti-rabbit-IgG conjugated to Alexa Fluor 568 (1:200, A10042, Invitrogen) and a goat anti-mouse-IgG conjugated to Alexa Fluor 488 (1:200, A11001, Invitrogen) were used as secondary antibodies.

For western blotting, we used a mouse monoclonal anti-phospho-p70 S6 Kinase (1:1000, 9206S, Cell Signaling), a rabbit monoclonal anti-human IRE1-p (1:1000, gift from Genentech (Chang et al., 2018a), a mouse monoclonal antibody anti NaK ATPase a5 (1:1000, deposited by Fambrough, D.M., DSHB), a rabbit monoclonal anti-phospho-HDAC4/HDAC5/HDAC7 (1:1000, 3424S, Cell Signaling), a rabbit monoclonal antibody anti-Phospho-eIF2α (Ser51) (1:1000, 9721S, Cell Signaling) and a mouse monoclonal anti-α-tubulin (1:2500, T5168, Sigma-Aldrich) followed by anti-rabbit-IgG and mouse-IgG antibodies coupled to HRP (1:2000, NA934, NA931, GE Healthcare).

### Immunofluorescence

For immunofluorescence, cells were fixed with 4% paraformaldehyde in PBS (pH 7.4) for 20 min. Cells were then washed three times with PBS and subsequently quenched by incubation in 50 mM NH_^4^_Cl in PBS for 5 min. Followed by permeabilization with 0.11% Triton X-100 for 5 min. Thereafter, cells were washed three times in PBS and blocked in PBS supplemented with 0.5% fish skin gelatin (G7765, Sigma-Aldrich) for 20 min. Cells were then incubated with the primary antibody (in blocking buffer) for 25 min, washed three times with blocking buffer and incubated with the secondary antibody (in blocking buffer) coupled to a fluorescent dye for 20 min. Cells on the coverslip were washed twice in milliQ water and dried for 3 min on a tissue with cells facing up. Finally, each coverslip with cells was mounted with Prolong antifade medium (+DAPI, P36935, Invitrogen) on a microscope slide. Samples were viewed with a Leica SPE confocal microscope using a 63× oil lens and 2× zoom.

### Quantification of Sec body formation

Cells positive for Sec bodies contain at least two large (>0.5 µm) (typically 3–10) round Sec16-positive structures, whereas the typical ERES pattern observed in Schneider's medium has largely disappeared (see Fig. 1A,A′) (Zacharogianni et al., 2014). For all conditions, immunofluorescence analysis was performed three times or more unless otherwise stated and at least four to five fields (∼25–30 cells per field) were recorded and analyzed per experiment. The response per field was determined by dividing the total amount of cells with Sec bodies by the total amount of cells. Finally, the average between all the fields was calculated and statistical analyses was performed using a one-tailed paired *t*-test.

### Cell diameter measurement

After incubation with different buffers, S2 cells were stained with phalloidin–TRITC 1: 4000 (P1951, Sigma-Aldrich) for 20 min to detect F-actin. To measure the cell cross-sectional diameter, confocal images of equatorial cell sections were taken. The diameter was drawn by hand and measured using the ‘Measure’ function of ImageJ.

### Cell fluorescence intensity measurement

To measure the Bip immunofluorescence intensity in the cells under different conditions, confocal images of full projections were analyzed. The measuring tool of ImageJ was used to measure the Bip immunofluorescence intensity cell by cell. The intensity in Schneider's medium was set as 1 and the intensity of the other conditions was expressed relative to that in Schneider's medium.

### XBP1/RIDD assay and *IRE1* PCR

10×10^6^ cells grown in Schneider's medium and 20×10^6^ cells incubated in KRB for 4 h were spun down (5 min at 200 ***g***) and washed in PBS-MilliQ prior to RNA extraction. For each condition, RNA was extracted using the RNeasy Mini Kit (Qiagen). The RNA concentrations were measured on the NanoDrop ND-1000 UV-Vis Spectrophotometer (Thermo Scientific). 1 μg RNA were used to synthesize cDNA using the GoScript Reverse Transcription System kit (Promega).

For each condition, a PCR was prformed using Taq polymerase (Promega) and visualized on agarose gel to assess *xbp1*, *bip, gadd45, sparc, indy, PIG-Wa*, *ire1* and *h2a* (control) PCR product. Primers used were: *xbp1* forward, 5′-CAGATGCATCAGCCAATCCAAC-3′; *xbp1* reverse, 5′-CACAACTTTCCAGAGTGAG-3′; *bip* forward, 5′-CTGCAGGTGATGCGCATCATCAA-3′; *bip* reverse, 5′-CTTGCCCTTCTTCTTCTTGTACAGCTTGA-3′; *gadd45* forward, 5′-ATGGTCGTCGAGGAGAACTGCAG-3′; *gadd45* reverse, 5′-CTCCAGCAGTACCTCGTGCATGT-3′; *indy* forward, 5′-CCATGAGCCTCAATACCAAATCGTTGGA-3′; *indy* reverse, 5′-TGTAGACCAACATGGATGGCACCG-3′; *PIG-Wa* forward, 5′-TTCTGGCTGTGGATTTCCCTTCGTATC-3′; *PIG-Wa* reverse, 5′-TTTCGTGAATCCCAGTATGAAGAAGGCATT-3′; *sparc* forward, 5′-GTCGGACTGCTCTGTGTATC-3′; *sparc* reverse, 5′-ATGGTCCTTGTTGGAGTCGC-3′; *h2a* forward, 5′-GTGGAAAAGGTGGCAAAGTGAA-3′; *h2a* reverse, 5′-TTCTTGTTGTCACGAGCAGCAT-3′; *ire1* forward, 5′-ATGAGAAGACGGACTGCACG-3′; and *ire1* reverse, 5′-GATCTGCTCGCCCTCCTTAC-3′.

### ROS detection assay

The stock solution of H2DCFDA (10 mM in DMSO) was diluted to 5 µM in Schneider's medium directly before usage. 10^6^ cells/ml were incubated with 5 µM H2DCFDA in Schneider's medium in the dark for 1 h (and up to 4 h) at 26°C in a 48-well plate. After incubation, the cells were washed three times with Schneider's medium to remove excess H2DCFDA that might be noncovalently associated with the extracellular leaflet of the plasma membrane. The cells were then incubated in the treatment medium (as described above) and the fluorescence intensity of the dye was immediately recorded over a period of 1 h at 26°C using a Spark multimode microplate reader (Tecan) with an excitation of 480 nm and an emission of 530 nm. The fluorescence intensity of five fields of cells per condition was measured every 5 min for a period of 1 h. Each experiment was performed at least three times or more.

The difference in DCF intensity for each condition was calculated by using the last value (1 h) minus the first value (0 h). The difference in intensity of Schneider's medium (usually very small, less than 10%) was used and set as the baseline at the value of 1. The difference in any other conditions was calculated as ‘Difference in DCF intensity ^(condition^ ^x)^/ Difference in DCF intensity (Schneider's medium)’. The fold difference was then expressed as a fold change over this baseline.

### Western blotting

A total of 4×10^6^ cells per condition were treated as described above. After treatment, the cells were harvested on ice and lysed in the following buffer [50 mM Tris-HCl pH 7.5, 150 mM NaCl, 1% Triton X- 100, 50 mM NaF, 1 mM Na_^3^_VO_^4^_, 25 mM Na_^2^_-β-glycerophosphate supplemented with a protease inhibitor tablet (11836153001, Roche)]. The lysates were cleared by centrifugation at 20,000 ***g*** for 20 min at 4°C. Supernatants were collected and the protein concentration was determined with a BCA protein assay kit (Thermo Fisher Scientific). 50 µg of protein was mixed with 5× SDS loading dye, boiled for 5 min and separated on an 8% SDS-PAGE gel. Then, separated proteins were transferred to a PVDF membrane. Hereafter, the PVDF membrane was blocked in TBS plus 0.05% Tween-20 and 5% BSA (Sigma-­­Aldrich) (blocking buffer). Primary antibodies were added to blocking buffer. After an overnight incubation at 4°C, the membrane was washed three times in TBS plus 0.05% Tween-20 over 45 min and incubated with secondary antibodies for 1 h at room temperature. The membrane was washed three times washing in TBS+0.05%Tween-20 and developed by enhanced chemiluminescence (Bio-Rad) with Image Quant™ LAS 4000.

### Cytoplasmic pH measurement

The cytoplasmic pH was estimated by using the ratiometric fluorescent GFP-mutant pHluorin2 (Mahon, 2011) attached to mCherry in a cell-based assay. Cloning of pHluorin2-mCherry was performed by amplifying the fragment out of pAG304GPD-ypHluorin2 [a gift from Simon Alberti, Biotechnology Center (BIOTEC), Center for Molecular and Cellular Bioengineering (CMCB), Technische Universität Dresden, Dresden, Germany] using forward primer, 5′-GAGGGTACCATGGTGAGCAAGGGCG-3′ and reverse primer, 5′-CGACTCGAGTTATTTGTATAGTTCATCCATGCCA -3′

The fragment was cloned into the pMT-V5 vector using KpnI and XhoI restriction sites. pMT-pHluorin2-mCherry was transfected in S2 cells using Effectene transfection reagent (Qiagen) with a 1:10 ratio of DNA to Effectene Reagent. Upon acidification, pHluorin2 excitation wavelength decreases, while the mCherry excitation signal remains largely unchanged. The ratio was calculated from the fluorescence intensity of pHluorin2 and mCherry determined using a Leica SP8 confocal microscope. The average ratio was calculated from 120 cells over five viewing fields. A pH calibration curve was made by incubating transfected cells for 30 min in buffers with a different pH supplemented with 75 µM monensin (M5273, Sigma-Aldrich), 10 µM nigericin (N7143, Sigma-Aldrich), 10 mM 2-deoxyglucose (D6134, Sigma-Aldrich) and 10 mM NaN_3_ (10325720, Thermo Fisher Scientific) to allow equilibration of the intracellular and extracellular pH. The ratio was calculated as described above.

### ATP measurement

To measure the ATP concentration, we used the CellTiter-Glo 2.0 kit (Promega) and followed the manufacturer’s instruction. In brief, S2 cells (8×10^4^) were plated in a transparent 96-well plate and treated as indicated above. Every treatment was performed at least in triplicate and each experiment was performed two or three times. To determine the intracellular ATP concentration, 100 μl of fresh incubation medium was added to the treated cells and a 0.5× volume CellTiter-Glo 2.0 (Promega) was added to each well. The background signal was determined by adding CellTiter-Glo 2.0 (Promega) to medium without cells. The total volume was transferred to a 96-well Greiner flat white plate. Luminescence was measured with a Tecan Spark multimode microplate reader (emission wavelength 560 nm).

To determine the extracellular ATP level, the incubation medium was transferred to a 96-well Greiner flat white plate and 0.5× volume CellTiter-Glo 2.0 (Promega) was added to each well. The luminescence was measured as above. An ATP calibration curve was made by measuring different known concentrations of ATP (Sigma-Aldrich) in Schneider's medium.

### Semi-intact cell assay

Wild-type S2 cells (1.5×10^6^) were plated on coverslips and permeabilized with 10 µg/ml digitonin (D141, Sigma-Aldrich) in KRB for 2 h at 26°C. Subsequently, cells were fixed and Sec bodies were visualized by immunostaining of Sec16. To test the effect of ATP, AMP or adenosine on Sec body formation, the semi-intact cells were incubated in the presence of 0.5 mM ATP (A1852, Sigma-Aldrich), 0.5 mM AMP (01930, Sigma- Aldrich) or 0.5 mM adenosine (A9251, Sigma-Aldrich). Note that the digitonin was not removed. The permeabilization efficiency was determined by using the non-membrane-permeable dye TO-PRO-3 iodide (T3605, Thermo Fisher Scientific) (Fig. 6A). The import buffer used in the SIC system was 20 mM HEPES, 110 mM KAc, 2 mM MgAc, 5 mM NaAc and 0.5 mM EGTA (pH was set at either 6.0 or 7.4 with KOH). For the RNA degradation experiment in the SIC system, we incubated S2 cells on coverslips for 2 h at 26°C in Schneider's medium containing 10 µg/ml digitonin (Sigma-Aldrich) with or without 0.25 U/ul RNase 1 (EN0602, Thermo Fisher Scientific).

### RNA sequencing

A total of 10×10^6^ S2 cells were incubated in KRB and in Schneider's medium for 4 h as described above. Two biological replicates were performed. Total RNA was isolated using the RNeasy mini kit (Qiagen). The Utrecht Sequencing Facility (USEQ) generated RNA libraries using a RiboZero (Illumina) and TruSeq Stranded Total RNA Library Prep kit (Illumina). Hereafter, libraries were sequenced on Illumina NextSeq500 1×75 bp high output. Read quality was checked using fastqc. Reads were mapped to the *Drosophila melanogaster* Dm6 reference genome (UCSC) using STAR (2.4.0.1) with default parameters (Dobin et al., 2013). We generated the read count per transcript table with an in-house Python2.7 script using pysam (https://github.com/pysam-developers/pysam) and the Dm6 annotation file (Ensembl). Differential expression between the conditions was determined using EdgeR (3.24.3) analysis in the R bioconductor package (Robinson et al., 2010). The DAVID web tool was used for GO analysis with default parameters (https://david.ncifcrf.gov/summary.jsp). The GEO accession number for the RNA-seq data reported in this paper is GSE143810.

### smFISH

Plated wild-type S2 cells were first treated with KRB (see above), and fixed and labeled for endogenous FMR1 or Sec16 as described above in the immunofluorescence section. After incubation with the secondary antibody, cells were washed three times with PBS and cells were post-fixed in 4% paraformaldehyde in PBS (pH 7.4) for 10 min. Following a washing three times in PBS, cells were further incubated for 5 min in 10% formamide (17899, Thermo Fisher Scientific) in DEPC-treated water. They were then incubated overnight on a droplet containing one fluorescent smFISH probe [125 nM in 1% dextransulfate (D8906, Sigma-Aldrich), 10% formamide in DEPC-treated water at 37°C] in a moistened chamber to avoid drying. Cells were washed twice for 30 min with 10% formamide in DEPC-treated water and mounted with Prolong antifade medium (plus DAPI) on a microscope slide. The TMR-oligo(dT)30× was purchased from IDT. A widefield Leica MM-AF microscope was used with a 100× lens for imaging.

### Statistics

Significance of the data was calculated and made by using GraphPad Prism 5.02. Statistical analyses were performed by using a one-tailed paired *t*-test and denoted *P<0.05, **P<0.01, ***P<0.001. Error bars represent s.e.m. except for Fig. 6B,C′, Figs S2B′ and S5A,A′,B where they represent s.d.

## Supplementary Material

Supplementary information

Reviewer comments

## References

[JCS258685C1] Aguilera-Gomez, A., van Oorschot, M. M., Veenendaal, T. and Rabouille, C. (2016). In vivo vizualisation of mono-ADP-ribosylation by dPARP16 upon amino-acid starvation. *eLife* 5, e21475. 10.7554/eLife.21475 27874829 PMC5127640

[JCS258685C2] Aulas, A., Fay, M. M., Lyons, S. M., Achorn, C. A., Kedersha, N., Anderson, P. and Ivanov, P. (2017). Stress-specific differences in assembly and composition of stress granules and related foci. *J. Cell Sci.* 130, 927-937. 28096475 10.1242/jcs.199240 PMC5358336

[JCS258685C3] Bah, A. and Forman-Kay, J. D. (2016). Modulation of intrinsically disordered protein function by post-translational modifications. *J. Biol. Chem.* 291, 6696-6705. 10.1074/jbc.R115.695056 26851279 PMC4807257

[JCS258685C4] Bounedjah, O., Hamon, L., Savarin, P., Desforges, B., Curmi, P. A. and Pastré, D. (2012). Macromolecular crowding regulates assembly of mRNA stress granules after osmotic stress: new role for compatible osmolytes. *J. Biol. Chem.* 287, 2446-2458. 10.1074/jbc.M111.292748 22147700 PMC3268405

[JCS258685C5] Boyce, M. and Yuan, J. (2006). Cellular response to endoplasmic reticulum stress: a matter of life or death. *Cell Death Differ.* 13, 363-373. 10.1038/sj.cdd.4401817 16397583

[JCS258685C6] Brett, C. L., Tukaye, D. N., Mukherjee, S. and Rao, R. (2005). The yeast endosomal Na+(K+)/H+ exchanger Nhx1 regulates cellular pH to control vesicle trafficking. *Mol. Biol. Cell* 16, 1396-1405. 10.1091/mbc.e04-11-0999 15635088 PMC551501

[JCS258685C7] Chang, T.-K., Lawrence, D. A., Lu, M. M., Tan, J., Harnoss, J. M., Marsters, S. A., Liu, P., Sandoval, W., Martin, S. E. and Ashkenazi, A. (2018a). Coordination between two branches of the unfolded protein response determines apoptotic cell fate. *Mol. Cell* 71, 629-636.e625. 10.1016/j.molcel.2018.06.038 30118681

[JCS258685C8] Chang, T. K., Lawrence, D. A., Lu, M., Tan, J., Harnoss, J. M., Marsters, S. A., Liu, P., Sandoval, W., Martin, S. E. and Ashkenazi, A. (2018b). Coordination between two branches of the unfolded protein response determines apoptotic cell fate. *Mol. Cell* 71, 629-636.e625. 30118681 10.1016/j.molcel.2018.06.038

[JCS258685C9] Clark, K., MacKenzie, K. F., Petkevicius, K., Kristariyanto, Y., Zhang, J., Choi, H. G., Peggie, M., Plater, L., Pedrioli, P. G., McIver, E. et al. (2012). Phosphorylation of CRTC3 by the salt-inducible kinases controls the interconversion of classically activated and regulatory macrophages. *Proc. Natl. Acad. Sci. U.S.A.* 42, 6986-6991. 10.1073/pnas.1215450109 PMC3479463 23033494

[JCS258685C10] Coelho, D. S., Cairrao, F., Zeng, X., Pires, E., Coelho, A. V., Ron, D., Ryoo, H. D. and Domingos, P. M. (2013). Xbp1-independent Ire1 signaling is required for photoreceptor differentiation and rhabdomere morphogenesis in Drosophila. *Cell reports* 5, 791-801. 10.1016/j.celrep.2013.09.046 24183663 PMC3858604

[JCS258685C11] Condon, K. J. and Sabatini, D. M. (2019). Nutrient regulation of mTORC1 at a glance. *J. Cell Sci.* 132, jcs222570. 10.1242/jcs.222570 31722960 PMC6857595

[JCS258685C12] Dobin, A., Davis, C. A., Schlesinger, F., Drenkow, J., Zaleski, C., Jha, S., Batut, P., Chaisson, M. and Gingeras, T. R. (2013). STAR: ultrafast universal RNA-seq aligner. *Bioinformatics* 29, 15-21. 10.1093/bioinformatics/bts635 23104886 PMC3530905

[JCS258685C13] Ducommun, S., Ford, R. J., Bultot, L., Deak, M., Bertrand, L., Kemp, B. E., Steinberg, G. R. and Sakamoto, K. (2014). Enhanced activation of cellular AMPK by dual-small molecule treatment: AICAR and A769662. *Am. J. Physiol. Endocrinol. Metab.* 306, E688-E696. 10.1152/ajpendo.00672.2013 24425763 PMC3948978

[JCS258685C14] Farhan, H., Wendeler, M. W., Mitrovic, S., Fava, E., Silberberg, Y., Sharan, R., Zerial, M. and Hauri, H. P. (2010). MAPK signaling to the early secretory pathway revealed by kinase/phosphatase functional screening. *J. Cell Biol.* 189, 997-1011. 10.1083/jcb.200912082 20548102 PMC2886346

[JCS258685C15] Gaddam, D., Stevens, N. and Hollien, J. (2012). Comparison of mRNA localization and regulation during endoplasmic reticulum stress in Drosophila cells. *Mol. Biol. Cell* 24, 14-20. 10.1091/mbc.e12-06-0491 23135994 PMC3530775

[JCS258685C16] Gomez-Navarro, N. and Miller, E. (2016). Protein sorting at the ER-Golgi interface. *J. Cell Biol.* 215, 769-778. 10.1083/jcb.201610031 27903609 PMC5166505

[JCS258685C17] Harrington, P. E., Biswas, K., Malwitz, D., Tasker, A. S., Mohr, C., Andrews, K. L., Dellamaggiore, K., Kendall, R., Beckmann, H., Jaeckel, P. et al. (2015). Unfolded protein response in cancer: IRE1α inhibition by selective kinase ligands does not impair tumor cell viability. *ACS Med. Chem. Lett.* 6, 68-72. 10.1021/ml500315b 25589933 PMC4291719

[JCS258685C18] Hetz, C. (2012). The unfolded protein response: controlling cell fate decisions under ER stress and beyond. *Nat. Rev. Mol. Cell Biol.* 13, 89-102. 10.1038/nrm3270 22251901

[JCS258685C19] Hollien, J. and Weissman, J. S. (2006). Decay of endoplasmic reticulum-localized mRNAs during the unfolded protein response. *Science* 313, 104-107. 10.1126/science.1129631 16825573

[JCS258685C20] Hollien, J., Lin, J. H., Li, H., Stevens, N., Walter, P. and Weissman, J. S. (2009). Regulated Ire1-dependent decay of messenger RNAs in mammalian cells. *J. Cell Biol.* 186, 323-331. 10.1083/jcb.200903014 19651891 PMC2728407

[JCS258685C21] Ivan, V., de Voer, G., Xanthakis, D., Spoorendonk, K. M., Kondylis, V. and Rabouille, C. (2008). Drosophila Sec16 mediates the biogenesis of tER sites upstream of Sar1 through an arginine-rich motif. *Mol. Biol. Cell* 19, 4352-4365. 10.1091/mbc.e08-03-0246 18614796 PMC2555954

[JCS258685C22] Jaitovich, A. and Bertorello, A. M. (2010). Intracellular sodium sensing: SIK1 network, hormone action and high blood pressure. *Biochim. Biophys. Acta* 1802, 1140-1149. 20347966 10.1016/j.bbadis.2010.03.009

[JCS258685C23] Jalihal, A. P., Pitchiaya, S., Xiao, L., Bawa, P., Jiang, X., Bedi, K., Parolia, A., Cieslik, M., Ljungman, M., Chinnaiyan, A. M. et al. (2020). Multivalent proteins rapidly and reversibly phase-separate upon osmotic cell volume change. *Mol. Cell* 79, 978-990.e975. 10.1016/j.molcel.2020.08.004 32857953 PMC7502480

[JCS258685C24] Joo, J. H., Wang, B., Frankel, E., Ge, L., Xu, L., Iyengar, R., Li-Harms, X., Wright, C., Shaw, T. I., Lindsten, T. et al. (2016). The noncanonical role of ULK/ATG1 in ER-to-golgi trafficking is essential for cellular homeostasis. *Mol. Cell* 62, 491-506. 27203176 10.1016/j.molcel.2016.04.020 PMC4993601

[JCS258685C25] Jwa, M. and Chang, P. (2012). PARP16 is a tail-anchored endoplasmic reticulum protein required for the PERK- and IRE1alpha-mediated unfolded protein response. *Nat. Cell Biol.* 14, 1223-1230. 10.1038/ncb2593 23103912 PMC3494284

[JCS258685C26] Kilchert, C., Weidner, J., Prescianotto-Baschong, C. and Spang, A. (2010). Defects in the secretory pathway and high Ca2+ induce multiple P-bodies. *Mol. Biol. Cell* 21, 2624-2638. 10.1091/mbc.e10-02-0099 20519435 PMC2912349

[JCS258685C27] Kim, J. and Guan, K.-L. (2019). mTOR as a central hub of nutrient signalling and cell growth. *Nat. Cell Biol.* 21, 63-71. 10.1038/s41556-018-0205-1 30602761

[JCS258685C28] Kondylis, V. and Rabouille, C. (2003). A novel role for dp115 in the organization of tER sites in Drosophila. *J. Cell Biol.* 162, 185-198. 10.1083/jcb.200301136 12876273 PMC2172793

[JCS258685C29] Korennykh, A. V., Egea, P. F., Korostelev, A. A., Finer-Moore, J., Zhang, C., Shokat, K. M., Stroud, R. M. and Walter, P. (2009). The unfolded protein response signals through high-order assembly of Ire1. *Nature* 457, 687-693. 10.1038/nature07661 19079236 PMC2846394

[JCS258685C30] Leung, A. K., Calabrese, J. M. and Sharp, P. A. (2006). Quantitative analysis of Argonaute protein reveals microRNA-dependent localization to stress granules. *Proc. Natl. Acad. Sci. USA* 103, 18125-18130. 10.1073/pnas.0608845103 17116888 PMC1838717

[JCS258685C31] Lopez-Hernandez, T., Puchkov, D., Krause, E., Maritzen, T. and Haucke, V. (2020). Endocytic regulation of cellular ion homeostasis controls lysosome biogenesis. *Nat. Cell Biol.* 22, 815-827. 10.1038/s41556-020-0535-7 32601373

[JCS258685C32] Mahon, M. J. (2011). pHluorin2: an enhanced, ratiometric, pH-sensitive green florescent protein. *Adv. Biosci. Biotechnol.* 2, 132-137. 10.4236/abb.2011.23021 21841969 PMC3152828

[JCS258685C33] Miesenböck, G., De Angelis, D. A. and Rothman, J. E. (1998). Visualizing secretion and synaptic transmission with pH-sensitive green fluorescent proteins. *Nature* 394, 192-195. 9671304 10.1038/28190

[JCS258685C34] Moore, K. A. and Hollien, J. (2012). The unfolded protein response in secretory cell function. *Annu. Rev. Genet.* 46, 165-183. 10.1146/annurev-genet-110711-155644 22934644

[JCS258685C35] Munder, M. C., Midtvedt, D., Franzmann, T., Nuske, E., Otto, O., Herbig, M., Ulbricht, E., Muller, P., Taubenberger, A., Maharana, S. et al. (2016). A pH-driven transition of the cytoplasm from a fluid- to a solid-like state promotes entry into dormancy. *eLife* 5, e09347. 10.7554/eLife.09347 27003292 PMC4850707

[JCS258685C36] Owen, I. and Shewmaker, F. (2019). The role of post-translational modifications in the phase transitions of intrinsically Disordered proteins. *Int. J. Mol. Sci.* 20, 5501. 10.3390/ijms20215501 PMC6861982 31694155

[JCS258685C37] Patel, A., Malinovska, L., Saha, S., Wang, J., Alberti, S., Krishnan, Y. and Hyman, A. A. (2017). ATP as a biological hydrotrope. *Science* 356, 753. 10.1126/science.aaf6846 28522535

[JCS258685C38] Peters, L. Z., Hazan, R., Breker, M., Schuldiner, M. and Ben-Aroya, S. (2013). Formation and dissociation of proteasome storage granules are regulated by cytosolic pH. *J. Cell Biol.* 201, 663-671. 10.1083/jcb.201211146 23690178 PMC3664706

[JCS258685C39] Petrovska, I., Nuske, E., Munder, M. C., Kulasegaran, G., Malinovska, L., Kroschwald, S., Richter, D., Fahmy, K., Gibson, K., Verbavatz, J. M. et al. (2014). Filament formation by metabolic enzymes is a specific adaptation to an advanced state of cellular starvation. *eLife* 3, e02409. 10.7554/eLife.02409.036 PMC4011332 24771766

[JCS258685C40] Rabouille, C. and Alberti, S. (2017). Cell adaptation upon stress: the emerging role of membrane-less compartments. *Curr. Opin. Cell Biol.* 47, 34-42. 10.1016/j.ceb.2017.02.006 28342303

[JCS258685C41] Robinson, M. D., McCarthy, D. J. and Smyth, G. K. (2010). edgeR: a Bioconductor package for differential expression analysis of digital gene expression data. *Bioinformatics* 26, 139-140. 10.1093/bioinformatics/btp616 19910308 PMC2796818

[JCS258685C42] Sprangers, J. and Rabouille, C. (2015). SEC16 in COPII coat dynamics at ER exit sites. *Biochem. Soc. Trans.* 43, 97-103. 10.1042/BST20140283 25619252

[JCS258685C43] Sunderhaus, E. R., Law, A. D. and Kretzschmar, D. (2019). ER responses play a key role in Swiss-Cheese/Neuropathy Target Esterase-associated neurodegeneration. *Neurobiol. Dis.* 130, 104520. 10.1016/j.nbd.2019.104520 31233884 PMC6690343

[JCS258685C44] Teesalu, M., Rovenko, B. M. and Hietakangas, V. (2017). Salt-inducible kinase 3 provides sugar tolerance by regulating NADPH/NADP+ redox balance. *Curr. Biol.* 27, 458-464. 10.1016/j.cub.2016.12.032 28132818

[JCS258685C45] Traut, T. W. (1994). Physiological concentrations of purines and pyrimidines. *Mol. Cell. Biochem.* 140, 1-22. 10.1007/BF00928361 7877593

[JCS258685C46] van Leeuwen, W. and Rabouille, C. (2019). Cellular stress leads to the formation of membraneless stress assemblies in eukaryotic cells. *Traffic* 20, 623-638. 10.1111/tra.12669 31152627 PMC6771618

[JCS258685C47] Walter, P. and Ron, D. (2011). The unfolded protein response: from stress pathway to homeostatic regulation. *Science* 334, 1081. 10.1126/science.1209038 22116877

[JCS258685C48] Wehr, M. C., Holder, M. V., Gailite, I., Saunders, R. E., Maile, T. M., Ciirdaeva, E., Instrell, R., Jiang, M., Howell, M., Rossner, M. J. et al. (2013). Salt-inducible kinases regulate growth through the Hippo signalling pathway in Drosophila. *Nat. Cell Biol.* 15, 61-71. 10.1038/ncb2658 23263283 PMC3749438

[JCS258685C49] Wein, M. N., Foretz, M., Fisher, D. E., Xavier, R. J. and Kronenberg, H. M. (2018). Salt-inducible kinases: physiology, regulation by cAMP, and therapeutic potential. *Trends Endocrinol. Metab.* 29, 723-735. 10.1016/j.tem.2018.08.004 30150136 PMC6151151

[JCS258685C50] Weiss, A., Charbonnier, E., Ellertsdottir, E., Tsirigos, A., Wolf, C., Schuh, R., Pyrowolakis, G. and Affolter, M. (2010). A conserved activation element in BMP signaling during Drosophila development. *Nat. Struct. Mol. Biol.* 17, 69-76. 10.1038/nsmb.1715 20010841

[JCS258685C51] Wilhelmi, I., Kanski, R., Neumann, A., Herdt, O., Hoff, F., Jacob, R., Preussner, M. and Heyd, F. (2016). Sec16 alternative splicing dynamically controls COPII transport efficiency. *Nat. Commun.* 7, 12347. 10.1038/ncomms12347 27492621 PMC4980449

[JCS258685C52] Yan, C., Yan, Z., Wang, Y., Yan, X. and Han, Y. (2014). Tudor-SN, a component of stress granules, regulates growth under salt stress by modulating GA20ox3 mRNA levels in Arabidopsis. *J. Exp. Bot.* 65, 5933-5944. 10.1093/jxb/eru334 25205572 PMC4203129

[JCS258685C53] Yan, C., Liu, J., Gao, J., Sun, Y., Zhang, L., Song, H., Xue, L., Zhan, L., Gao, G., Ke, Z. et al. (2019). IRE1 promotes neurodegeneration through autophagy-dependent neuron death in the Drosophila model of Parkinson's disease. *Cell Death Dis.* 10, 800. 10.1038/s41419-019-2039-6 31641108 PMC6805898

[JCS258685C54] Zacharogianni, M., Aguilera-Gomez, A., Veenendaal, T., Smout, J. and Rabouille, C. (2014). A stress assembly that confers cell viability by preserving ERES components during amino-acid starvation. *eLife* 3, e04132. 10.7554/eLife.04132 PMC4270098 25386913

[JCS258685C55] Zhang, X., Lv, H., Zhou, Q., Elkholi, R., Chipuk, J. E., Reddy, M. V., Reddy, E. P. and Gallo, J. M. (2014). Preclinical pharmacological evaluation of a novel multiple kinase inhibitor, ON123300, in brain tumor models. *Mol. Cancer Ther.* 13, 1105-1116. 10.1158/1535-7163.MCT-13-0847 24568969 PMC4013241

