# Supplementary material for: Activation of IRE1, PERK and salt-inducible kinases leads to Sec body formation in Drosophila S2 cells
Source: J Cell Sci. 2021 Sep 6;134(17):jcs258685. doi: 10.1242/jcs.258685 (PMC8445602; doi:10.1242/jcs.258685)
Supplement: Supplementary information [file joces-134-258685-s1.pdf]

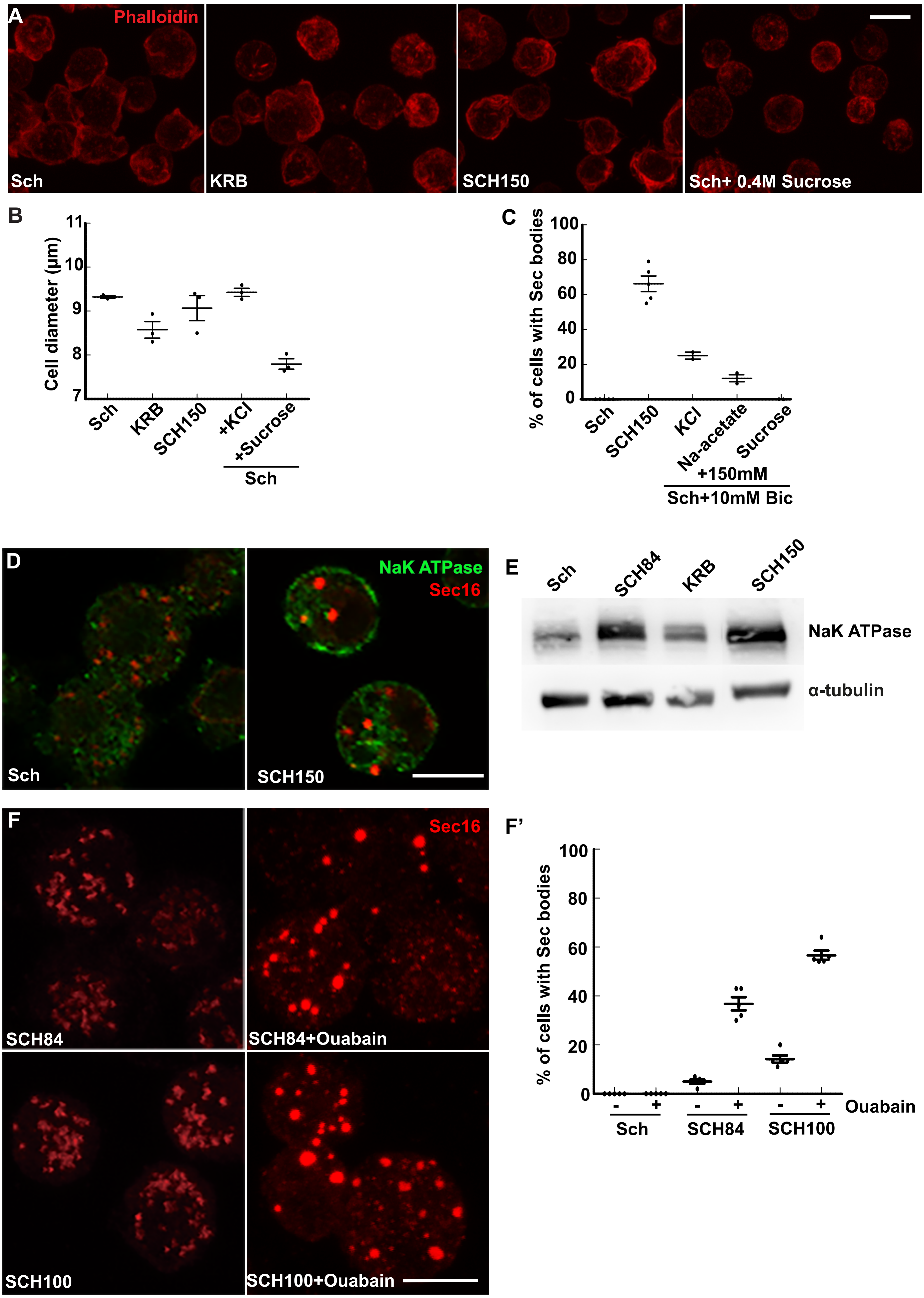
A
Phalloidin
Sch
KRB
SCH150
Sch+ 0.4M Sucrose
B
Cell diameter (µm)
11
10
9
8
7
Sch
KRB
SCH150
+KCl
+Sucrose
Sch
C
% of cells with Sec bodies
100
80
60
40
20
0
Sch
SCH150
KCl
Na-acetate
Sucrose
+150mM
Sch+10mM Bic
D
NaK ATPase
Sec16
Sch
SCH150
E
Sch
SCH84
KRB
SCH150
NaK ATPase
α-tubulin
F
Sec16
SCH84
SCH84+Ouabain
SCH100
SCH100+Ouabain
F'
% of cells with Sec bodies
100
80
60
40
20
0
-
+
Ouabain
Sch
SCH84
SCH100

Fig. S1. High salt stress is not equivalent to osmotic shock

A: Visualization of the cell perimeter by Phalloidin staining (A) in cells incubated in Schneider's (Sch), KRB and SCH150 for 4h at 26°C as well as Sch+0.4M sucrose for 1.5h at 26°C.

B: Measurement (in µm) of the cell diameter after incubations in conditions mentioned on the panel. Note that incubation in 0.4M sucrose leads to a 22% decrease of the cell diameter.

C: Quantification of Sec body formation (marked by Sec16) in cells incubated in Sch and Schneider's supplemented with 10mM sodium bicarbonate and 150mM of NaCl (SCH150), KCl, Na-acetate, and sucrose for 4h at 26°C. Note that the strong Sec body formation response is specific for addition of 150mM NaCl.

D: IF visualization of the NaK ATPase (green) and Sec16 (red) in cells incubated in Sch and SCH150 for 4h at 26°C. Note the strong increase of NaK ATPase on the cell plasma membrane after incubation in SCH150.

E: Western blot of S2 cells extract after incubation in Sch, SCH84, KRB and SCH150 for 4h at 26°C stained for NaK ATPase and α-tubulin. Note that the NaK ATPase level increased after incubation in SCH150.

F, F': IF visualization (F) and quantification (F') of Sec body formation (marked by Sec16) in cells incubated in Sch, SCH84 and SCH100 supplemented by ouabain (1µM) for 4h at 26°C.

Scale bar: 10µm.

Errors bars: SEM

Journal of Cell Science • Supplementary information

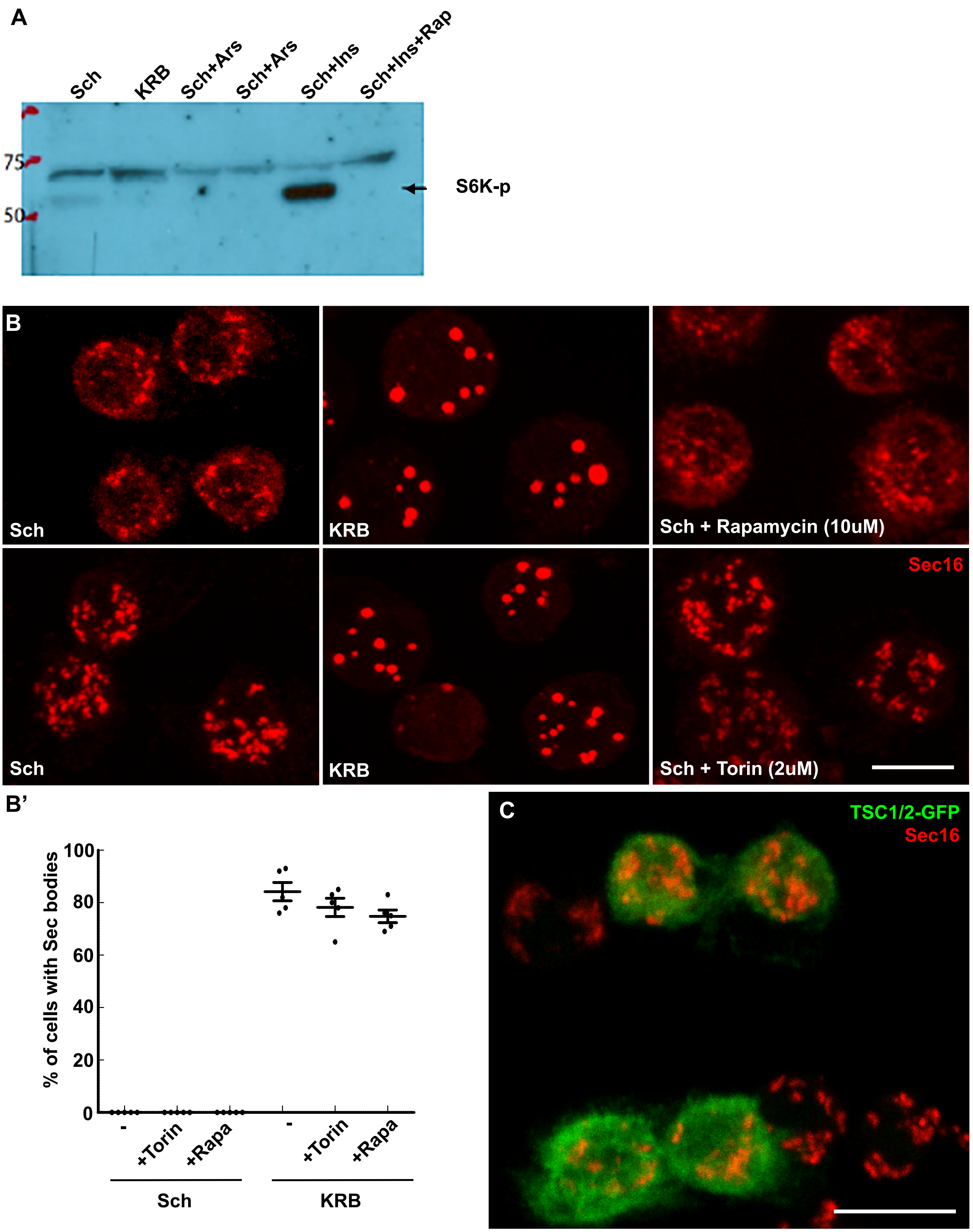
A
Sch
KRB
Sch+Ars
Sch+Ars
Sch+Ins
Sch+Ins+Rap
75
50
S6K-p
B
Sch
KRB
Sch + Rapamycin (10uM)
Sec16
Sch
KRB
Sch + Torin (2uM)
B'
% of cells with Sec bodies
100
80
60
40
20
0
-
+Torin
+Rapa
-
+Torin
+Rapa
Sch
KRB
C
TSC1/2-GFP
Sec16

Fig. S2. TORC1 inhibition does not lead to Sec body formation

A: Western blot of extract of S2 cells extract incubated as mentioned and stained for S6K-p. This shows that S6K is no longer phosphorylated after KRB incubation for 4h and that rapamycin inhibits S6K-p stimulated by insulin.

B, B': IF visualization (B) and quantification (B') of Sec body formation (marked by Sec16) in cells grown in Schneider's (Sch) also supplemented by mTORC1 inhibitors Rapamycin (10µM) and Torin (2µM) for 4h at 26°C. Note that the sole inhibition of mTORC1 does not lead to Sec body formation contrary to incubation in KRB.

C: IF Visualization of Sec16 in cells overexpressing the mTORC1 inhibitor TSC1/2-GFP. Note that Sec bodies do not form in these transfected cells.

Scale bar: 10µm

Error bars: SD

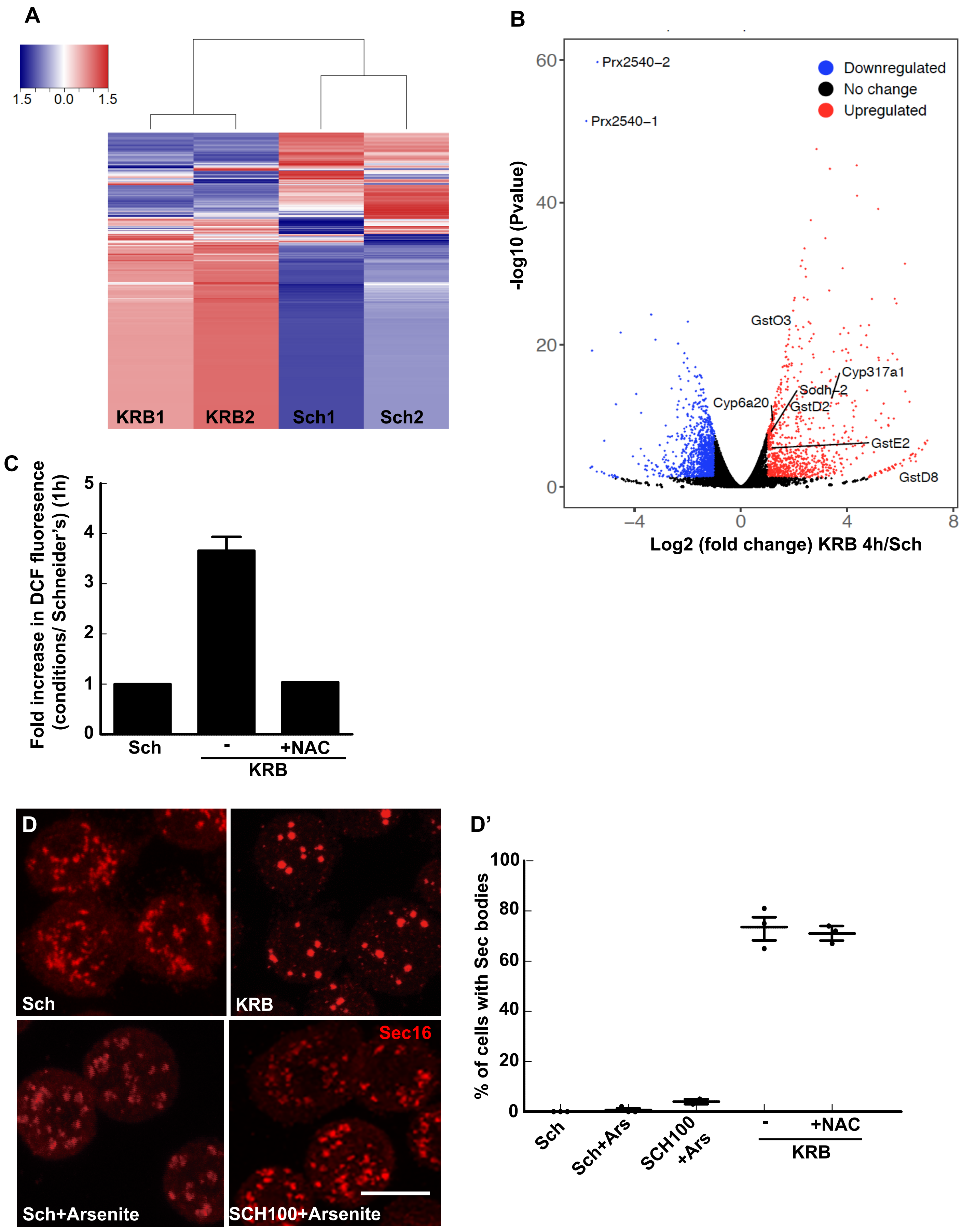

Journal of Cell Science • Supplementary information

Fig. S3. KRB incubation leads to oxidative stress but oxidative stress does not lead to Sec body formation

A: Heatmap generated from all detected transcripts highlighting the similarities of the number of reads between the conditions based on the Euclidean distance. The replicates of each condition clusters together.

B: Volcano plot mRNAs identified by RNA sequencing that are differentially modulated between Schneider's and KRB.

C: Graph showing the increase in DCF fluorescence measuring the production of ROS upon KRB for 4h at 26°C when compared to Schneider's (Sch). Sec body formation is quantified in D'. Note that addition of N-acetyl-L-cysteine (NAC) suppresses ROS production but has no effect on Sec body formation.

D, D': IF visualization (D) and quantification (D') of Sec body formation in cells in Schneider's supplemented with sodium arsenite (Ars, 2.5mM) and SCH100+Ars (2.5mM) for 4h at 26°C. Note that none of these conditions elicit Sec body formation.

Scale bar: 10µm

Errors bars: SEM

Note that ROS production even when combined with moderate salt stress, does not lead to Sec body formation. Conversely, inhibiting the ROS production in KRB with N-acetyl cysteine (NAC, Suppl Figure S3C-D'), does not change the Sec body formation response.

Journal of Cell Science • Supplementary information

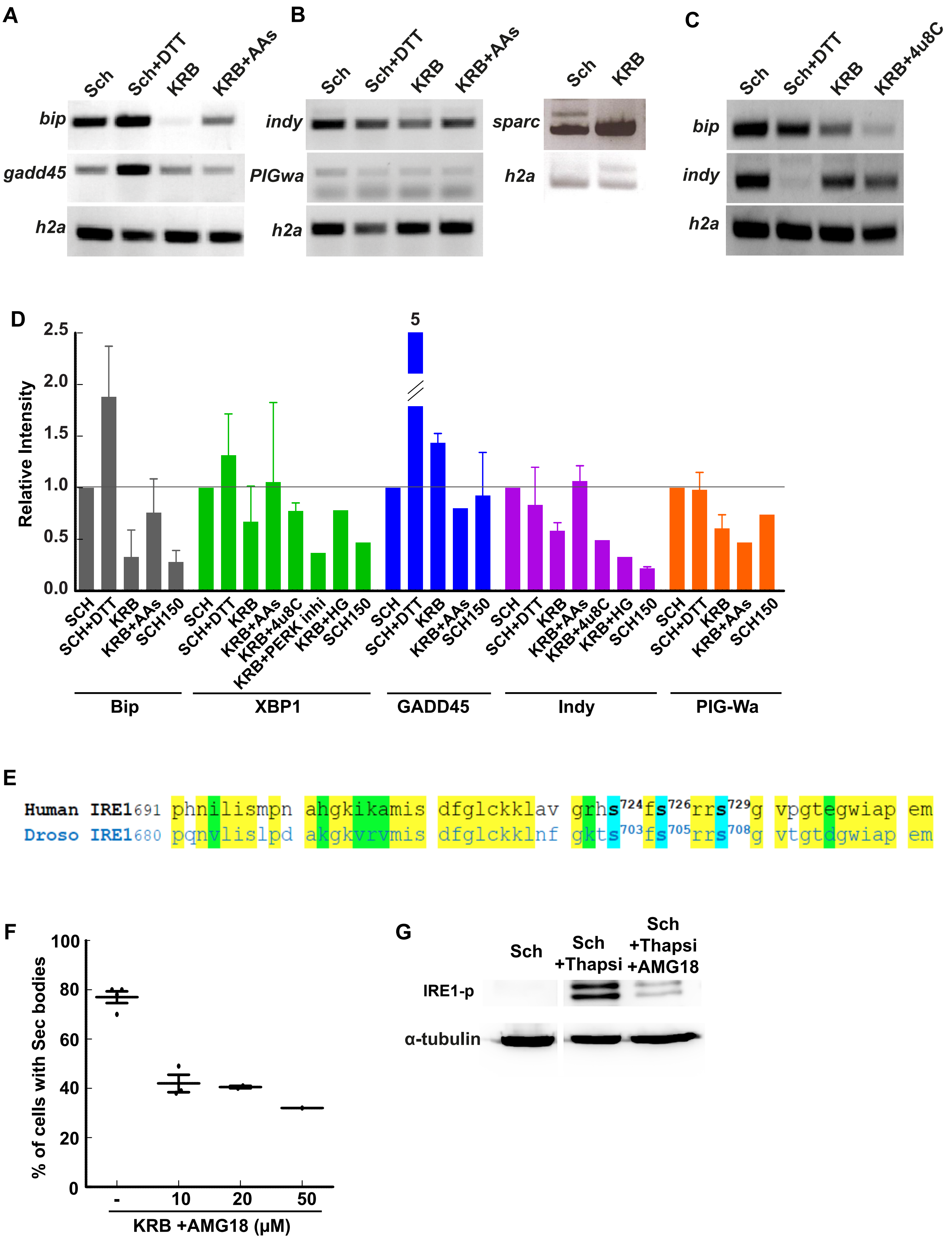

Journal of Cell Science • Supplementary information

Fig. S4. Drosophila IRE1 and specificity and efficacy of AMG18

A: Visualization of the PCR products of UPR *bip*, *gadd45*, and mRNAs from cells grown in the conditions mentioned in the panel for 4h at 26°C with *h2a* as a control.

B: Visualization of the PCR RIDD products of *indy*, *PIGwa* and *sparc* from cells grown in the conditions mentioned in the panel for 4h at 26°C with *h2a* as a control.

C: Visualization of the PCR products of *bip* and *indy* from cells in KRB supplemented or not with 4u8C for 4h at 26°C.

D: Quantification of the PCR results upon different conditions mentioned. Normalized to Schneider's (set at 1).

E: Comparison of the IRE1 catalytic sites sequences in human (H) and *Drosophila* (D). The green highlights the identity in and around the kinase site (underlined). The serine (S) in blue is the auto-phosphorylated site. Note that the two other serines (in blue) are also conserved.

F: Quantification of Sec body formation (marked by Sec16) in cells incubated in KRB supplemented by 10, 20 and 50µM AMG18. Note that the inhibition of Sec body formation is very similar for each concentration (about 45%).

G: Western blot of S2 cells extract after their incubation in Schneider's supplemented with Thapsigargin (2µM) and AMG18 (10µM) using the IRE1-p specific antibody and an α-tubulin antibody. Note that the incubation with AMG18 inhibits IRE1-p by 84%.

Errors bars: SEM

Journal of Cell Science • Supplementary information

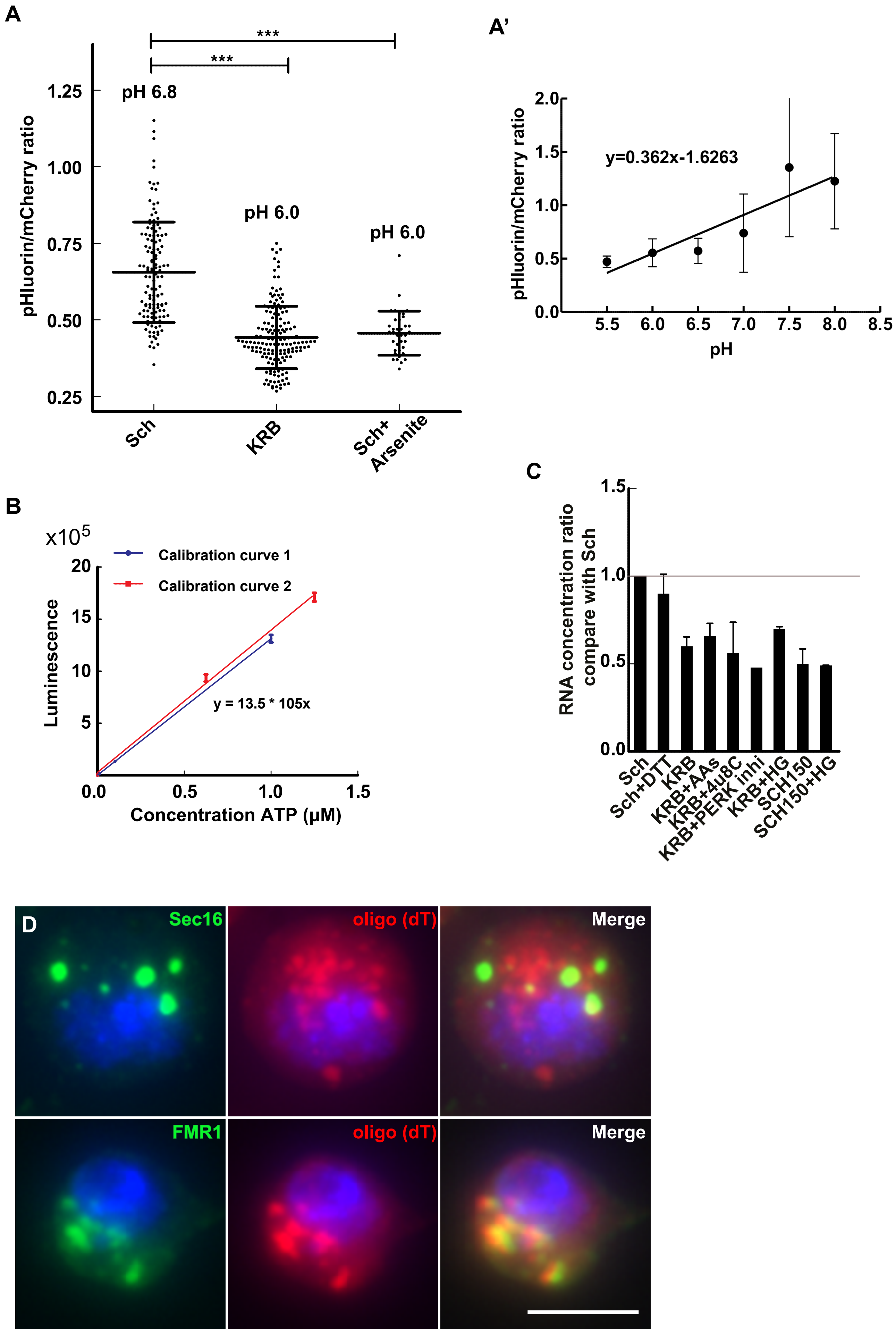

Journal of Cell Science • Supplementary information

Fig. S5. pH measurements, calibration curves and oligo(dT) smFISH

A, A': Measurement of the intracellular pH (using the pHluorin-mCherry- fusion protein) in cells incubated in Schneider's (Sch), KRB and Sch+0.5mM arsenite (A). pH calibration curve, plotting the ratio between pHluorin and mCherry in buffers with a different pH (A')

B: ATP concentration calibration curve, plotting the ATP concentration versus the luminescence intensity.

C: RNA concentration after extraction from the conditions mentioned in the panel when compared with Schneider's (Sch) (set as 1).

D: Single molecule FISH using oligo(dT) probes (red) on cells incubated in KRB for 4h and labeled for Sec16 (green, to mark Sec bodies, and with FMR1 (green) to mark stress granules). Note that as expected polyA tailed RNAs co-localize with stress granules but not with Sec bodies.

Scale bar: 10µm

Error bars: SD (A, A', B), SEM(C).

Table S1 (related to Suppl Figure S3). List of genes up and down regulated upon KRB incubation. Differential gene expression between 4h starved (KRB) and 4h fed (Schneider's) using EdgeR. The gene name, log fold change, P-value and FDR are shown. Significant (Empirical Bayes p<0.05) up regulated genes are highlighted in red and down regulated genes in blue.

Click here to download Table S1

Table S2. List of drugs with providers and concentrations

Chemicals	Suppliers	Article number	Stock concentrations	Solvant	Final concentrations
Adenosine	Sigma-Aldrich	A9251	10mM	MilliQ	0.5mM
AICAR	Sigma-Aldrich	A9978	100mM	MilliQ	1mM
Amino-acids solution	Sigma-Aldrich	R7131	228.7mM	KRB	5.72mM
AMP (Adenosine 5′-monophosphate disodium salt)	Sigma-Aldrich	01930	100mM	MilliQ	0.5mM
AMG18 (IRE1 kinase activity)	Gift from Genentech		10mM	DMSO	10µM<br>20µM<br>50µM
APS (Ammonium persulfate)	Sigma-Aldrich	A7460	1M	MilliQ	500µM
Arsenite (Na (meta) arsenite)	Sigma-Aldrich	S7400	0.5M	MilliQ	2.5mM
ATP (Adenosine 5′-triphosphate disodium salt hydrate)	Sigma-Aldrich	A1852	100mM	MilliQ	0.5mM
CCCP (Carbonyl cyanide 3-chlorophenylhydrazone)	Sigma-Aldrich	C2759	100mM	DMSO	25µM
Dasatinib	Sigma-Aldrich	SML2589	80mM	DMSO	20µM
2-Deoxy-D-glucose	Sigma-Aldrich	D6134	1M	MilliQ	20mM
DTT (Dithiothreitol)	Biorad	161-0611	2M	DMSO	5mM
Dorsomorphin	Sigma-Aldrich	P5499	1mM	DMSO	1µM
H_2O_2 (Hydrogen peroxide)	Sigma-Aldrich	H1009	2M	MilliQ	1mM
HG-9-91-01 (pan SIK inhibitor)	MedChemExpress/Bio-Connect	HY-15776	5mM	DMSO	5µM
KCl (Potassium Chloride)	Sigma-Aldrich	P9541	2M	MilliQ	150mM
Ionomycin from Streptomyces conglobatus	Sigma-Aldrich	I9657	2.8mM	DMSO	2.8µM
Na-acetate (Sodium acetate)	Fisher Scientific	S/2040/53	2M	MilliQ	150mM
N-Acetyl-L-cysteine	Sigma-Aldrich	A7250	30mM	MilliQ	300µM
NaCl (Sodium Chloride)	J.T.Baker	0278	2M	MilliQ	84mM,<br>100mM,<br>150mM
ON123300	Selleckchem	S8161	10mM	DMSO	10µM
Ouabain octahydrate	Sigma-Aldrich	O3125	50mM	MilliQ	1µM
PERK inhibitor I	Sigma-Aldrich	516535	10mM	DMSO	5µM
Rapamycin	Sigma-Aldrich	R0395	10mM	DMSO	10µM
RNase I	ThermoFisher	EN0602	10U/µl		0.25U/µl
SB203580	Sigma-Aldrich	S8307	30mM	DMSO	30µM
Sucrose	Sigma-Aldrich	S0389	2M	MilliQ	0.4M
Thapsigargin	Sigma-Aldrich	SML1845	1mM	DMSO	2µM
Torin	Invivogen	inh-tor1	3mM	DMSO	2µM
4u8C (IRE1 nuclease activity Inhibitor)	Merck Millipore	412512	122mM	DMSO	30µM

Journal of Cell Science • Supplementary information
