# Supplementary material for: Activation of IRE1, PERK and salt-inducible kinases leads to Sec body formation in Drosophila S2 cells
Source: J Cell Sci. 2021 Sep 6;134(17):jcs258685. doi: 10.1242/jcs.258685 (PMC8445602; doi:10.1242/jcs.258685)
Supplement: Reviewer comments [file joces-134-258685_review_history.pdf]

Activation of IRE1, PERK and salt-inducible kinases leads to Sec body formation in *Drosophila* S2 cells

Chujun Zhang, Wessel van Leeuwen, Marloes Blotenburg, Angelica Aguilera-Gomez, Sem Brussee, Rianne Grond, Harm H. Kampinga and Catherine Rabouille
DOI: 10.1242/jcs.258685

Editor: David Stephens

Review timeline

Submission to Review Commons:	16 January 2021
Submission to Journal of Cell Science:	22 March 2021
Editorial decision:	25 March 2021
First revision received:	19 July 2021
Accepted:	21 July 2021

Reviewer 1

Evidence, reproducibility and clarity

In this manuscript, Zhang and colleagues document the mechanisms by which, in Drosophila cells, phase separation of the non-membrane bound Sec bodies do occur. The manuscript is well written, the topic is really new and the data presented are generally convincing. This manuscript identifies two novel regulators of Sec bodies formation with the necessary and sufficient role of Salt-induced kinases and the conditional role of IRE1 thereby providing

Major points

1) Salt inducible kinases belongs to the AMPK family and previous literature has literature has linked AMPK signaling to the activation of ER stress and IRE1. How do the authors reconcile their results with these observations? Would the authors predict that pan-AMPK inhibitors (e.g. Dorsomorphin and ON123300) exert similar effects than HG-9-91-01?

2) AICAR is an agonist of AMPK also reported to act on SLKs, and in addition it has been shown that AICAR is also capable of modulating ER stress (e.g. PMID: 27847321) and more generally proteostasis (e.g. PMID: 21315436). In the experimental conditions described by the authors, would AICAR be sufficient or combined with AA deprivation to drive Sec bodies formation?

3) The authors show that under their experimental conditions, the activation of both IRE1 kinase (phosphorylation) and RNase (XBP1 mRNA splicing) activity occurs but they do not link this observation to the effects even though they use AMG18 (which behaves as a Kinase Inhibiting RNase Attenuator). The ability of this compound to alter IRE1 activity should be fully documented and the authors should also use other types of inhibitors directly targeting the RNase domain (e.g. 4u8c or MKC compounds which are commercially available), but again their effects on drosophila IRE1 should be properly documented.

4) If IRE1 RNase is required for the formation of Sec bodies and the latter contain RNA, do the authors think that IRE1 activation might selectively cleave several RNA through RIDD activity (obviously not SPARC) which could in turn change the local concentration of RNA (as well as their nature) thereby favoring Sec body formation?

© 2021. Published by The Company of Biologists under the terms of the Creative Commons Attribution License (https://creativecommons.org/licenses/by/4.0/).

5) A direct functional link between IRE1 and AMPK was previously reported, it would be great if the authors could evaluate whether this is conserved between SLK and IRE1 in their experimental settings.
6) If IRE1 is activated by the UPR, then what is the activation status of the other arm of the UPR (PERK and ATF6)? And are those other arm involved as well? If not do could the author speculate on the existence of some selective mechanisms that could only make sec body formation dependent on IRE1

7) At last, how do the authors link IRE1 activation to ATP depletion?

Minor points

1) The scheme indicates that the UPR is a required step in the IRE1 mediated formation of Sec bodies but this is not absolutely demonstrated in the manuscript

2) Phospho-IRE1 blots could be reprobed with anti IRE1 antibodies (perhaps by testing several polyclonal anti IRE1 antibodies raised against the whole cytosolic domain of mammalian IRE1) to ensure that the expression levels of the protein are not affected (cytosolic domains of human and dm IRE1 exhibit >50% identity)

3) Are the anti-phosphoIRE1 antibodies used in this study directed towards P-S724 (of the human sequence)? The phosphorylation site recognized by this antibodies should be indicated in the Materials & Methods section.

Significance

This is the first report that describe a role of SLKs and IRE1 in the formation of non membrane bound organelles and as such is of great interest.

It provides another functional link between the AMPK family of kinase and ER stress signaling and demonstrates that it is connected to a biological outcome never reported before.

the audience is the cell biology community at large expanding toward metabolic regulations my expertise: regulation of ER proteostasis

Referees cross-commenting

I agree with reviewer 2 regarding a simple control that should be added
"To argue for a direct causative relationship, they should at least examine whether amino acid addition suppresses UPR (IRE1 activity) in KRB."

this issue is very important as noted by reviewer 2 as well
"The authors should determine the activity of the other pathways in UPR/ER stress (ATF6 and PERK) and test their roles in Sec body formation."

Reviewer 2

Evidence, reproducibility and clarity

In this work, the authors utilized Drosophila S2 cell culture to identify the molecular pathways triggered by a starvation medium that cause the formation of Sec body. The authors assessed several candidate pathways by manipulating cell culture conditions and quantifying Sec body formation. They found that 1) high NaCl concentration (~200mM) was sufficient to induce Sec body, mechanistically relying on activating salt-inducible kinases (SIKs) rather than osmotic stress; 2) amino acid starvation triggered Unfolded Protein Response (UPR), which was necessary to Sec body formation under medium NaCl stress but insufficient when elicited alone; 3) intracellular ATP decrease had similar effect as UPR on Sec body formation and thus was speculated as a mechanistic link between UPR and Sec body assembly.

Major comments:

© 2021. Published by The Company of Biologists under the terms of the Creative Commons Attribution License (https://creativecommons.org/licenses/by/4.0/).

1. In the working model (Fig. 8) the authors proposed both high and medium salt stress activated SIKs, but no strong evidence showed SIKs were activated with medium salt concentration. Based on HDAC phosphorylation result (Fig 2B), SIKs seems only activated in SCH150 medium (high salt) but not much different in KRB medium when compared to Schneider medium, thus unlikely activated in SCH84/100 as well. HG only had a limited effect in KRB, SCH100-DTT (Fig 3B, 5B). The authors should better quantify the SIKs activity in various medium-salt media (KRB/SCH84/SCH100), using p-HDAC or other known targets. It is more likely that medium salt turned on a SIK-independent pathway that has to collaborate with amino acid starvation to induce Sec body.
2. To our knowledge, there are only two Drosophila SIKs, sik2 and sik3, instead of three SIKs stated in the manuscripts. In S2 cells, sik2 is barely expressed and sik3 expression is relatively low as well (based on Flybase expression data). Thus it is feasible to perform knockdown or over-expression of sik3 to directly assess its involvement in Sec body formation.
3. Authors concluded that amino acid starvation triggered UPR but there is no direct evidence supporting this argument. Authors only showed that amino acid suppressed Sec body formation (Fig 3A) and DTT, potentially through inducing UPR, promoted Sec body formation (Fig 5A'). To argue for a direct causative relationship, they should at least examine whether amino acid addition suppresses UPR (IRE1 activity) in KRB.
4. IRE pathway appears to be not the only downstream effector of KRB starvation medium that led to Sec body formation, as IRE1 inhibition did not fully recapitulate the effect of amino acid addition in KRB (Fig 5B). The authors should determine the activity of the other pathways in UPR/ER stress (ATF6 and PERK) and test their roles in Sec body formation. Even if the other pathways are not involved, they should prove the sufficiency of IRE1 pathway by over-expressing constitutively active IRE1 mutant under SCH100 condition so that other complex effects from DTT/aa-starvation can be excluded.
5. What's the possible mechanism of ATP on Sec body formation? Does ATP directly affect the behavior of Sec16/23/24 or somehow suppress the ER stress? Authors should examine the UPR markers in the ATP depletion/add-back experiment.

Minor points:

1. Authors should note in the main text the actual Na+ concentration in SCH84/100/150. These numbers are very misleading about the actual concentration.
2. KRB and KRB21 were used interchangeably, which caused great confusion. For some experiments, the main text stated that it was performed with KRB but it was labeled as KRB21 in the figure (Fig 6B), or the opposite (Fig 3A').
3. Western blot in Fig 1E and 2B should be labeled with quantification as the loading controls varied a lot.

Significance

This work is a follow-up study on the previous discovery of Sec body in S2 cells by Rabouille group. The authors identified two potential molecular pathways (SIKs and UPR) responsible for Sec body formation but the work is not sufficient to elucidate mechanistically how the two pathway lead to Sec body formation. More importantly, as the Sec body formation has only been observed in S2 cell culture under specific starving condition (Zacharogianni et. al., 2014), we question the relevance of the these finding to the study in vivo or in other organisms. The fact that Sec16 and Sec24 have IDRs and capability of phase separating into droplets is interesting and suggests a phase separation-dependent regulation on the secretory pathway. The work, however, did not address how these pathway affect phase separation of Sec16/24 and how secretory pathway is affected.

The reviewers have a background in fly genetics, RNA granules and phase separation study. We could be unaware of the impact of the study on those who study stress response or secretory pathway.

© 2021. Published by The Company of Biologists under the terms of the Creative Commons Attribution License (https://creativecommons.org/licenses/by/4.0/).

Original submission

First decision letter
MS ID#: JOCES/2021/258685

MS TITLE: Salt Inducible Kinase activation and IRE1-dependent intracellular ATP depletion to form Sec bodies in Drosophila cells

AUTHORS: Catherine Rabouille, Chujun Zhang, Wessel va Leeuwen, Marloes Blotenburg, Angelica Aguilera-Gomez, Sem Brussee, Rianne Grond, and Harm Kampinga
ARTICLE TYPE: Research Article

Thanks for submitting your manuscript via Review Commons. I have evaluated the manuscript along with the reviews, your detailed response to them, and your revision plan and would like to invite you to submit a revised version. I consider the major comments to be valid, especially around AMPK and the role of IRE1. Your revision plan seems entirely appropriate. The only thing I might add for your to consider would relate to the ADP/ATP ratio. This clearly has major implications for AMPK activation. I appreciate that this was not raised by the reviewers but I mention it in the context of your proposed revision for consideration. Given the extent of revisions proposed, I may also seek the opinion of the original reviewers upon submission of the revised manuscript.

The reviewers' reports and a copy of this decision letter, are available at: https://submit-jcs.biologists.org and click on the 'Manuscripts with Decisions' queue in the Author Area. (Corresponding author only has access to reviews.)

We are aware that you may be experiencing disruption to the normal running of your lab that makes experimental revisions challenging. If it would be helpful, we encourage you to contact us to discuss your revision in greater detail. Please send us a point-by-point response indicating where you are able to address concerns raised (either experimentally or by changes to the text) and where you will not be able to do so within the normal timeframe of a revision. We will then provide further guidance. Please also note that we are happy to extend revision timeframes as necessary.

Please ensure that you clearly highlight all changes made in the revised manuscript. Please avoid using 'Tracked changes' in Word files as these are lost in PDF conversion.

I should be grateful if you would also provide a point-by-point response detailing how you have dealt with the points raised by the reviewers in the 'Response to Reviewers' box. Please attend to all of the reviewers' comments. If you do not agree with any of their criticisms or suggestions please explain clearly why this is so.

First revision

Author response to reviewers' comments

Note: The authors have not included the preliminary version of their response to the reviewers.

We thank the reviewers from Review Commons (passed to Journal of Cell Science) for their thorough reading of our manuscript, for their constructive criticisms, comments and important suggestions.
Please find below the full point-by-point rebuttal to all of them.

We have experimentally addressed the 13 main points of the reviewers plus one raised by the editor. These findings are summarized in the revision plan and changes are incorporated in the revised manuscript when appropriate.

In a null shell, we have shown that:

© 2021. Published by The Company of Biologists under the terms of the Creative Commons Attribution License (https://creativecommons.org/licenses/by/4.0/).

-AMPK family members are unlikely to be involved in Sec body formation, except for SIK as we originally proposed.

-IRE1 phosphorylation is partly activated by the absence of amino-acids in the starvation medium KRB, and that KRB incubation triggers the activation of the UPR.
-PERK is also activated by KRB and is necessary for Sec body formation.

Overall, we confirm that IRE1 activation is necessary for KRB induced Sec body formation but not sufficient. Sec body formation also needs PERK activation, but this is also not sufficient.
IRE1 and PERK activation needs to be combined to a moderate salt stress that activates SIKs. Therefore, we propose that either IRE1 and PERK activation potentiates the moderate salt stress, or that salt stress activates IRE1 and PERK.
Noticeably, we confirm that a strong SIK activation alone (through high salt stress) is enough to form Sec bodies.

We propose the following model
-Strong salt stress activates SIKs (strong inhibition by HG and no evidence of other kinase involved) and this is enough to drive Sec body formation.

KRB activates 3 pathways:
-Activation of IRE1 by AA starvation. IRE1 is less activated when AAs are present in the medium. IRE1 activation is necessary for Sec body formation (4u8C, AMG18) but not sufficient (Sch+DTT, constitutive active IRE1).
-Activation of PERK. Inhibition of PERK decreased Sec body formation upon KRB.
-Activation of SIK by moderate salt stress (partial HG inhibition). A moderate SIK activation alone is not enough to form Sec bodies. This needs to be combined with IRE1 and PERK activation.

What is common between KRB and SCH150?
-One common factor between KRB and SCH150 is the activation of the UPR (measured as the increase of Bip protein level). This activation in KRB is through IRE1 and PERK activation.
How UPR activation leads to Sec body formation has not been addressed in detail here.

-The ATP decrease in the cytoplasm appears to be a strong factor leading to Sec body formation in KRB. However, SCH150 incubation does not lead to this decrease.
One of the reviewers asked us to establish the link between IRE1 and the ATP decrease. We originally had experimental evidences that suggested that IRE1 activity (leading to the UPR activation) was upstream of this ATP decrease. However, we failed to confirm this. Furthermore, we now show that decreasing ATP with CCCP leads to UPR activation. This suggests that the ATP decrease leads to IRE1 (and PERK) activation.
In KRB, this initial ATP decrease could be due to the malfunctioning of mitochondria due to the oxidative stress imposed by KRB.

-Last, we have observed that both KRB and SCH150 incubation led to RNA degradation, suggesting that this could be pivotal to Sec body formation. However, we have not obtained experimental evidence to sustain this statement.

Reviewer #1 (Evidence, reproducibility and clarity (Required)):
In this manuscript, Zhang and colleagues document the mechanisms by which, in Drosophila cells, phase separation of the non-membrane bound Sec bodies do occur.
The manuscript is well written, the topic is really new and the data presented are generally convincing. This manuscript identifies two novel regulators of Sec bodies formation with the necessary and sufficient role of Salt-induced kinases and the conditional role of IRE1 thereby providing

Major points
Rev1.1: Salt inducible kinases belongs to the AMPK family and previous literature has linked AMPK signaling to the activation of ER stress and IRE1. How do the authors reconcile their results with these observations? Would the authors predict that pan-AMPK inhibitors (e.g. Dorsomorphin and ON123300) exert similar effects than HG-9-91-01?
>> We thank the reviewer for this suggestion of to test whether the salt stress (moderate in KRB

© 2021. Published by The Company of Biologists under the terms of the Creative Commons Attribution License (https://creativecommons.org/licenses/by/4.0/).

and strong in SCH150) could activate kinases of the AMPK family, other than SIKs.

Indeed, KRB induced Sec body formation is inhibited by 38% by the SIK Pan inhibitor HG9-91- 09 (HG), but HDAC4 (a known SIK target) does not appear to be phosphorylated. This is strikingly different from SCH150, where HDAC4 phosphorylation increases 4-fold when compared to Schneider's alone, and is strongly inhibited (97%) by HG. This correlates very well with the 80% inhibition of Sec body formation. We take it as a strong evidence that the salt stress in SCH150 activates SIKs that play an important role in Sec body formation. We experimentally confirmed this conclusion by showing that neither the AMPK pan-inhibitor Dorsomorphin (at 1 and 10μM) nor the more specific inhibitor ON123300 (10 and 25μM) did inhibit SCH150 induced Sec body formation (**revision plan point 1**, **Figure 2C**)

Regarding KRB, it is therefore possible that KRB activates other AMPK family members that are not SIKs. As HG has also been shown to inhibit P38 and Src, as above, we tested the role of specific inhibitors on KRB induced Sec body formation but we demonstrated no effect.

To test whether other members of the AMPK family members other than SIKs could be involved, we have incubated cells in KRB in the presence Dorsomorphin and ON123300 as above (**revision plan point 1**). However, again, neither Dorsomorphin (from 1-20uM) nor ON123300 affects KRB induced Sec body formation (**Figure 2C**).

Our conclusion for KRB is that the moderate salt stress applied by this incubation activates SIKs. The reason for which we cannot demonstrated HDAC4 phosphorylation remains uncertain. Either HDAC4 protein is largely degraded upon KRB, or HDAC4 is not a SIK target in cells incubated for KRB.

Rev1.2: AICAR is an agonist of AMPK also reported to act on SLKs, and in addition it has been shown that AICAR is also capable of modulating ER stress (Nie et al., 2017) and more generally proteostasis (Tang et al., 2011). In the experimental conditions described by the authors, would AICAR be sufficient or combined with AA deprivation to drive Sec bodies formation?
>> We have also performed the converse experiment of activating AMPK with AICAR (**revision plan point 2**). We used cells grown in Schneider's (full medium) but also in Schneider's that does not contain the AAs ("Sch buffer", that is meant to activate the AA starvation pathway through IRE1, see below). None of these conditions lead to Sec body formation, confirming that AMPK is not involved in Sec body formation (revised **Figure 2C**).

(see also editor point at the end of the rebuttal).

Rev1. 3: The authors show that under their experimental conditions, the activation of both IRE1 kinase (phosphorylation) and RNase (*xbp1* mRNA splicing) activity occurs but they do not link this observation to the effects even though they use AMG18 (which behaves as a Kinase Inhibiting RNase Attenuator). The ability of this compound to alter IRE1 activity should be fully documented and the authors should also use other types of inhibitors directly targeting the RNase domain (e.g. 4u8C or MKC compounds which are commercially available), but again their effects on drosophila IRE1 should be properly documented.
>> We thank the reviewer for this suggestion. We have performed experiment with the inhibitor of IRE1 nuclease activity, 4u8C, and found that it very strongly inhibits Sec body formation upon KRB, but not significantly upon SCH150 (high salt). This indicates that the IRE1 nuclease activity is required for this cellular response following IRE1 kinase autophosphorylation and activation of its nuclease activity. This has been included to the revised manuscript (**revision plan point 3**, revised **Figure 3E**)

As for properly documenting AMG18 in Drosophila cells, we kindly point the reviewer to the original and present *Suppl Figure S4* where we have shown 3 things: That the kinase site in the Drosophila IRE1 is very similar to that of human IRE1; that we have used AMG18 at the correct low concentration; and that it attenuates/inhibits thapsigargin induced IRE1 autophosphorylation by 84%. To the best of our knowledge, these results therefore show that AMG18 work just as well in Drosophila cells as it does in human cells.

Rev1. 4: If IRE1 RNase is required for the formation of Sec bodies and the latter contain RNA, do the authors think that IRE1 activation might selectively cleave several RNA through RIDD activity (obviously not SPARC) which could in turn change the local concentration of RNA (as well as their nature) thereby favoring Sec body formation?

© 2021. Published by The Company of Biologists under the terms of the Creative Commons Attribution License (https://creativecommons.org/licenses/by/4.0/).

>> The reviewer is correct that we have “dismissed” RIDD on the basis on *sparc* RNA whose level is not changing in KRB.

We have now reassessed whether RIDD is activated during KRB incubation. To do this, we first used our RNA Seq data (as shown in the (*Suppl Figure S3A and Suppl Table S1*) of cells in growing medium Schneider’s and KRB. We retrieved the level of expression of the validated and potential RIDD targets. Out of the 60 targets identified (Gaddam et al., 2012; Hollien and Weissman, 2006; Moore and Hollien, 2012), 21 are also downregulated in KRB (versus Sch, see **Table for reviewer at the end of this document**), suggesting that his RIDD might be stimulated in KRB. Note, however, that *Sparc*, *Hydr2*, *Tsp42E*, *MIpple* 2 and *Jagunal* are not.

To confirm that RIDD could be stimulated in KRB downstream of IRE1 activation, we performed PCR of *indy* and *PIGwa* (**revision plan point 4**), and found that both are indeed less expressed in KRB (about 30% less) (*Suppl Fig S4B, D*). However, this decrease is not rescued by incubation with 4u8C (*Suppl Fig S4C, D*), suggesting that the degradation is not via RIDD. Note, though, that it is rescued by the addition of AAs to KRB. These results suggest that RIDD is unlikely to be stimulated.

Furthermore, we observed the same (some stronger) decrease of these RNA level in SCH150. Interestingly, known UPR targets, such as *bip* and *xbp1* RNAs also appear degraded in KRB, (see also Rev 1.8) and this is also not rescued by 4u8C, but, as above, is by the addition of AAs.

Taken together, KRB incubation induces a general RNA degradation that is unlikely to be RIDD. Considering the inhibition in Sec body formation observed by both AMG18 and 4u8C (**Figure 3E**), we conclude that IRE1 has a pivotal role, but not through RIDD.

The reviewer suggests that Sec bodies are RNA based and would form either to protect RNAs that escape degradation. To begin to answer this question, we performed an RNA in situ by single molecule FISH using a poly(dT) using (**revision plan point 5**, *Suppl Figure S5D*). However, we found that Sec bodies are not labeled at all with this probe, whereas stress granules (that are another type of phase separated assemblies driven by stress and known to contain full length mRNAs) are very strongly positive (*Suppl Figure S5D*). This suggests that Sec bodies do not contain a high amount of polyA-tail containing mRNAs and are therefore unlikely to protect them from degradation.

Another possibility that Sec body form in response to a local increase in RNA degradation that could act as a template. Therefore, we used our incubated semi-intact S2 cells in Schneider’s in which Sec bodies do not form and added with RNase 1 to induce RNA degradation, to test whether Sec body formation would be induced. However, this was not the case. Last, we added the total RNA (extracted from KRB treated cells) to semi-intact S2 cells in Schneider’s and tested whether this would induce Sec body formation. Again, this was not the case. Altogether, although the hypothesis was attractive (also because RNA degradation also occurs in SCH150), we were not able to confirm this experimentally.

Rev1.5: A direct functional link between IRE1 and AMPK was previously reported, it would be great if the authors could evaluate whether this is conserved between SLK and IRE1 in their experimental settings.

>> We thank the reviewer for this suggestion. SLK is STE20 like-kinase (MST4, CG4527 in Drosophila). However, due to lack of evidence linking AMPK to Sec body formation (Rev1.1 and 1.2 above and editor point below), we have not investigated this further.

Rev1.6: If IRE1 is activated by the UPR, then what is the activation status of the other arm of the UPR (PERK and ATF6)? And are those other arms involved as well? If not do could the author speculate on the existence of some selective mechanisms that could only make sec body formation dependent on IRE1.

>>We thank the reviewer for this suggestion (**revision plan point 7**). We have now tested whether PERK is involved in KRB induced Sec body formation using PERK depletion by RNAi and PEK inhibition with the PERK inhibitor (GSK2606414 at 5uM).

As expected, the PERK inhibitor does not influence SCH150 induced Sec body formation (that we have shown largely depends on SIKs, see Rev1.1 and **revision plan point 1**) (not shown. However, it leads to a 50% inhibition in Sec body formation upon KRB, similar to the inhibition observed with AMG18. Furthermore, the combined IRE1 and PERK inhibition (at least with 4u8C) combined, inhibits KRB induced Sec bodies further than when the inhibitors are used alone(**Figure 4E**). This suggests that both IRE1 and PERK are activated upon KRB incubation and act in parallel in Sec

© 2021. Published by The Company of Biologists under the terms of the Creative Commons Attribution License (https://creativecommons.org/licenses/by/4.0/).

body formation.

Regarding ATF6, its depletion by RNAi does not affect Sec body formation (Figure 4E). Of note, manipulation of PERK and ATF6 did not alter Sec body formation induced by SCH150, showing that it relies on SIK activation (not shown).

Rev1.7: At last, how do the authors link IRE1 activation to ATP depletion?
>>This is an interesting point. We had originally proposed that activation of IRE1 (and possibly other kinases) would could lead to ATP depletion. This was shown by using AMG18 whose addition abolished the observed decrease in ATP concentration. However, we were not able to reproduce this result with 4u8C. Furthermore, we showed that a decrease in the cytoplasmic concentration of ATP (incubation with CCCP) appears upstream of the UPR and possibly IRE1. Therefore, we have revised the text and our conclusions on that aspect. (see also rev2.5)

Minor points
Rev 1.8: The scheme indicates that the UPR is a required step in the IRE1 mediated formation of Sec bodies but this is not absolutely demonstrated in the manuscript.
>>This is an excellent point. Addressing it has led to complex answers. Indeed, we have shown that in KRB, IRE1 is activated by auto-phosphorylation leading to *xpb1* RNA splicing.

However, it is correct that we have not shown that the UPR is activated. Here we did it by assessing the increase in expression of the known UPR target BIP protein upon KRB incubation (**revision plan point 8**). As expected, addition of DTT leads to a 4-fold increase, and KRB incubation to a 2.3-fold increase, suggesting that it stimulates the UPR.

We also monitored the expression of *bip* mRNA by PCR, as well as 2 other UPR mRNAs targets, *gadd45*, and *xbp*1. However, we could not demonstrate any significant increase in the RNA level of these targets. This is in contrast to the addition of DTT that leads to their significant increase, modest for *bip* and *xbp1* (about 65%) and strong for GADD45 (400%) (*Suppl Fig S4D*).

Instead, as mentioned above (Rev1.3), we find that the *xbp1* and *bip* RNA level decreases upon KRB, as much as the level of *Indy* and *PIGwa* (*Suppl Fig S4B, D*).

Taken together, these results suggest that the UPR is activated in KRB incubated cells but that due to the degradation of UPR RNA targets observed in KRB (not via RIDD), the UPR activation is modest.

However, we confirmed that IRE1 activation is necessary for Sec body formation as the inhibition of its kinase and nuclease activity results in a significant inhibition of their formation (69% in the case of 4u8C) (see Rev1.4, **revision plan point 4**, **Figure 3E**). PERK activity is also necessary and their combined inhibition both inhibits the observed UPR and largely Sec body formation.

Importantly, IRE1 activation (and UPR stimulation) alone is not enough to trigger Sec body formation as DTT addition to Schneider's does not lead to their formation. Our model is that Sec body formation requires SIK activation in a manner that we still do completely understood, though it appears to involve IRE1 as we observed a low level of *xbp1* in high salt. However, high salt driven Sec body formation is not sensitive to 4u8C, rendering the link complex.

Rev1.9: Phospho-IRE1 blots could be reprobed with anti IRE1 antibodies (perhaps by testing several polyclonal anti IRE1 antibodies raised against the whole cytosolic domain of mammalian IRE1) to ensure that the expression levels of the protein are not affected (cytosolic domains of human and dm IRE1 exhibit >50% identity).
>>This has been done but was unsuccessful. As presented originally, we have bypassed this by testing the *ire1* mRNA level and show that it does not change (**Figure 3B**). Furthermore (**revision plan point 9**).

Rev1.10: Are the anti-phosphoIRE1 antibodies used in this study directed towards P-S724 (of the human sequence)? The phosphorylation site recognized by this antibody should be indicated in the Materials & Methods section.
>>This antibody can detect the human IRE1 kinase activation loop where phosphorylation of Ser724, Ser726 and Ser729 takes place. In Drosophila they are Ser703, Ser705 and Ser708.

Human: LCKKLAVGRHS^{724}FS^{726}RRS^{729}GV (Chang et al., 2018)

Droso: LCKKLNFGKTS^{703}FS^{705}RRS^{708}GV (Yan et al., 2019)

© 2021. Published by The Company of Biologists under the terms of the Creative Commons Attribution License (https://creativecommons.org/licenses/by/4.0/).

We will specify this in the revised manuscript.

Significance (Required)
Rev1.11: This is the first report that describe a role of SLKs and IRE1 in the formation of non- membrane bound organelles and as such is of great interest.

It provides another functional link between the AMPK family of kinase and ER stress signaling and demonstrates that it is connected to a biological outcome never reported before.

The audience is the cell biology community at large expanding toward metabolic regulations.
>>Thank you

Referees cross-commenting
Rev1.12: I agree with reviewer 2 regarding a simple control that should be added

"To argue for a direct causative relationship, they should at least examine whether amino acid addition suppresses UPR (IRE1 activity) in KRB."
>>See Rev2.3.

Rev1.13: This issue is very important as noted by reviewer 2 as well
"The authors should determine the activity of the other pathways in UPR/ER stress (ATF6 and PERK) and test their roles in Sec body formation."
>>We agree. See Rev2.4 And Rev1.6.

Reviewer #2 (Evidence, reproducibility and clarity (Required)):
In this work, the authors utilized Drosophila S2 cell culture to identify the molecular pathways triggered by a starvation medium that cause the formation of Sec body. The authors assessed several candidate pathways by manipulating cell culture conditions and quantifying Sec body formation. They found that 1) high NaCl concentration (~200mM) was sufficient to induce Sec body, mechanistically relying on activating salt-inducible kinases (SIKs) rather than osmotic stress; 2) amino acid starvation triggered Unfolded Protein Response (UPR), which was necessary to Sec body formation under medium NaCl stress but insufficient when elicited alone; 3) intracellular ATP decrease had similar effect as UPR on Sec body formation and thus was speculated as a mechanistic link between UPR and Sec body assembly.

Major points
Rev 2.1: In the working model (Fig 8) the authors proposed both high and medium salt stress activated SIKs, but no strong evidence showed SIKs were activated with medium salt concentration. Based on HDAC phosphorylation result (Fig 2B), SIKs seems only activated in SCH150 medium (high salt) but not much different in KRB medium when compared to Schneider medium, thus unlikely activated in SCH84/100 as well. HG only had a limited effect in KRB, SCH100-DTT (Fig 3B, 5B). The authors should better quantify the SIKs activity in various medium-salt media (KRB/SCH84/SCH100), using p-HDAC or other known targets. It is more likely that medium salt turned on a SIK-independent pathway that has to collaborate with amino acid starvation to induce Sec body.
>>We thank the reviewer for this suggestion.
We agree that we could not demonstrate HDAC4 phosphorylation in cells incubated in KRB (see also Rev 2.8). However, the pan SIKs inhibitor inhibits 38% of the KRB induced Sec body formation. As a control, we showed that SIKs are activated by SCH150 as HDAC4 is strongly phosphorylated (4-fold when compared to Schneider's) and this phosphorylation is 97% inhibited by HG, in agreement with its effect on Sec body formation (80% inhibition).

It is therefore possible that, in cells incubated in KRB, HG inhibits other kinases, such as P38, Src, and AMPK family members. We therefore tested the effects on KRB induced Sec body formation of inhibiting P38 and Src with their specific inhibitors (**Figure 2C**), and we also tested AMPK inhibition using Dorsomorphin (compound C) at different concentrations and ON123300. However, none of these inhibitors had an effect on Sec body formation either triggered by KRB or by SCH150 (as expected since that it depends on SIKs).

Furthermore, Sec bodies did not form upon AMPK stimulation by the agonist AICAR (see also Rev1.1 and 1.2, **revision plan points 1 and 2**).

Taken together, we ruled out that AMPK is involved in Sec body formation in KRB (and

© 2021. Published by The Company of Biologists under the terms of the Creative Commons Attribution License (https://creativecommons.org/licenses/by/4.0/).

SCH150). Because of the lack of pharmacological evidences, we have not overexpressed AMPK (as suggested in **revision point 10**).

We therefore conclude that KRB activates SIKs, but that because the level of HDAC4 is very low in KRB, it makes the demonstration of its phosphorylation difficult.

Rev2.2: To our knowledge, there are only two Drosophila SIKs, sik2 and Sik3, instead of three SIKs stated in the manuscripts. In S2 cells, sik2 is barely expressed and sik3 expression is relatively low as well (based on Flybase expression data). Thus, it is feasible to perform knockdown or over-expression of sik3 to directly assess its involvement in Sec body formation.
>>We thank the reviewer for this suggestion. So far, we have overexpressed SIK2 and 3, but this manipulation on its own did not lead to Sec body formation, even in moderate salt stress. It also did not increase Sec body formation in cells under stress conditions (KRB and SCH150). As the result was negative, we have not incorporated this data in the manuscript, and this has not prompted us to perform their depletion.

Rev 2.3: Authors concluded that amino acid starvation triggered UPR but there is no direct evidence supporting this argument.
>>This is correct and we thank the reviewer for pointing this to us. Addressing this point has led to an unexpected level of complexity and generates more questions.

We have now tested whether KRB leads to the activation of the UPR in two ways (see Rev 1.4 above). First, we monitored the level of Bip protein by immunofluorescence and found that Bip level increased 2.2-fold in cells incubated in KRB when compared to Schneider's (and 4-fold in the presence of DTT, as expected) (**Figure 3D, D'**). This suggests that the KRB incubation stimulates the UPR.

We also monitored the level of *bip* mRNA as well as two other UPR mRNAs targets, *GADD45* and *xbp1* itself. As expected, their level increased upon DTT, but none increased in KRB incubation. In fact, both *bip* and *xbp1* RNA levels decreased as much as RIDD targets (see Rev 1.4, *Suppl Fig S4B, D*). This degradation is however unlikely to be via RIDD because addition of 4u8C did not rescued this degradation. We suggest that KRB stimulates an RNA degradation pathway that tempers the UPR stimulation. Interestingly, RNA degradation was also observed in SCH150.

Authors only showed that amino acid suppressed Sec body formation (Fig 3A) and DTT, potentially through inducing UPR, promoted Sec body formation (Fig 5A'). To argue for a direct causative relationship, they should at least examine whether amino acid addition suppresses UPR (IRE1 activity) in KRB.
>>We thank the reviewer for this other excellent point. We now show that the absence of AAs in KRB triggers IRE1-p activation, at least partially. Indeed, addition of AAs to KRB partially prevents IRE1-p (**point11**), and *xbp1* splicing (from 40%s/60%u in KRB alone compared to 28%s/72%u in KRB+AA (**Figure 3A, C**). Interestingly, addition of AAs rescued RNA degradation, at least *bip* and *indy*.

We conclude that the absence of AAs partly activates IRE1 and the UPR, but the effect is not very strong. PERK is also likely activated by the absence of AAs, and KRB also exerts a moderate salt stress that is instrumental to Sec body formation (and UPR activation). The absence of AAs in the medium decreases the tolerance of cells to salt stress. This suggests that there is an interplay between IRE1, PERK and SIK, that IRE1 and PERK are the modulator of salt stress or vice versa.

Of note, the observed RNA degradation is not via RIDD because it is not rescued by 4u8C. Instead, we propose that this RNA degradation is triggered by salt stress (also observed in SCH150)

Rev2.4: IRE1 pathway appears to be not the only downstream effector of KRB starvation medium that led to Sec body formation, as IRE1 inhibition did not fully recapitulate the effect of amino acid addition in KRB (Fig 5B).
The authors should determine the activity of the other pathways in UPR/ER stress (ATF6 and PERK) and test their roles in Sec body formation.
>>We thank the reviewer for this suggestion (**revision plan point 7**). We have now tested whether PERK (PEK in Drosophila) is involved in KRB induced Sec body formation using PEK depletion by RNAi and PEK inhibition through PERK inhibitor (GSK2606414).

As expected, the PERK inhibitor does not influence SCH150 induced Sec body formation

© 2021. Published by The Company of Biologists under the terms of the Creative Commons Attribution License (https://creativecommons.org/licenses/by/4.0/).

(that we have shown largely depends on SIKs, see Rev1.1 and **revision plan point 1**). However, it leads to a 50% inhibition in Sec body formation upon KRB, similar to the inhibition observed with AMG18. When combined, these inhibitors do not lead to a stronger inhibition of KRB induced Sec bodies (**Figure 4E**). This suggests that both IRE1 and PERK are activated upon KRB incubation and act in parallel in Sec body formation.

Regarding ATF6, its depletion by RNAi does not affect Sec body formation (**Figure 4E**).

Even if the other pathways are not involved, they should prove the sufficiency of IRE1 pathway by over-expressing constitutively active IRE1 mutant under SCH100 condition so that other complex effects from DTT/aa-starvation can be excluded.

>>IRE1 activity appears necessary for Sec body formation but not necessary. Indeed, it activation with adding DTT to Schneiders' is not enough to trigger Sec body formation.

To confirm this and address he reviewer's query, we have generated a constitutively active IRE1 where the 3 serines of the Drosophila catalytic sited (Ser703, 705 and 708, corresponding to mammalian IRE1 at S724, Ser726 and Ser729 have been mutated to aspartic acids (see Rev1.10 and *Suppl Figure S4E*)(Chang et al., 2018; Yan et al., 2019). Overexpression of this mutated IRE1 did not lead to Sec body formation under any conditions tested (Schneider's, SCH buffer, SCH100), showing that IRE1 activation is not sufficient (**Figure 4B, C**).

We have now convincingly showed that PERK activation is also necessary as well as a moderate salt stress likely via SIKs.

Rev2.5: What's the possible mechanism of ATP on Sec body formation? Does ATP directly affect the behavior of Sec16/23/24 or somehow suppress the ER stress? Authors should examine the UPR markers in the ATP depletion/add-back experiment.

>> The reviewer asks whether low ATP affects the dynamics Sec16 and the other COPII subunits in such a way that they form Sec bodies. In normal growing conditions where cytoplasmic ATP is about 2mM, Sec16 and other COPII subunits remains at ER exit sites. In low ATP conditions, Sec bodies form perhaps because as an hydrotrope, ATP maintains IDR proteins and Sec16 in particular "soluble" (not phase separated). We have discussed this in the original manuscript and kept this in the revised version. This has not been further tested because the in vitro manipulation of purified Sec16 is beyond the scope of this study (see Rev2.9)

Regarding the relation between low ATP and IRE1 activation: We had originally indeed proposed that activation of IRE1 (and other kinases) would be upstream of the drop in the ATP concentration. This was because this drop was inhibited in the presence of AMG18. However, we were not able to reproduce this result with 4u8C. Furthermore, we instead show that artificially lowering the cytoplasmic ATP concentration by poisoning mitochondria with CCCP triggers the UPR (**revision plan point 13**) (**Figure 3D'**). Consequently, we place the drop in ATP upstream of at least IRE1 and PERK. We discuss the issue in the revised manuscript.

Minor points

Rev 2.6: Authors should note in the main text the actual Na+ concentration in SCH84/100/150.These numbers are very misleading about the actual concentration.

>>We apologize with the confusion. We genuinely thought that the experimental set up would be easier to follow by the reader when we mentioned how much more NaCl was added to the growing medium (such as SCH150 meaning Sch+150mM NaCl). The actual concentration in Na^+ are mentioned in the Table 1 of the original and present manuscript.

As requested, we will also mention them in the Results section.

Rev2.7: KRB and KRB21 were used interchangeably, which caused great confusion. For some experiments, the main text stated that it was performed with KRB but it was labeled as KRB21 in the figure (Fig 6B), or the opposite (Fig 3A').

>>The addition of 21 mM of KCl in KRB makes it slightly more potent to form Sec bodies but the differences are not significant. We have unified the naming of these two buffers as KRB.

Rev2.8: Western blot in Fig 1E and 2B should be labeled with quantification as the loading controls varied a lot.

>> This has been done and is now added as **Figure 1D**, **revision point 14**)

Significance

© 2021. Published by The Company of Biologists under the terms of the Creative Commons Attribution License (https://creativecommons.org/licenses/by/4.0/).

Rev2.9: This work is a follow-up study on the previous discovery of Sec body in S2 cells by Rabouille group. The authors identified two potential molecular pathways (SIKs and UPR) responsible for Sec body formation but the work is not sufficient to elucidate mechanistically how the two pathways lead to Sec body formation.
>>We agree that we still do not understand the mechanistic details how Sec body form downstream of the signaling pathways involved. This requires Sec16 (and likely its small domain called SRCD we have identified in (Aguilera-Gomez et al., 2016)), but what happens to this protein domain in response to IRE1 activation and/or salt stress is not (and will not be) elucidated in this manuscript.

Rev2.10: More importantly, as the Sec body formation has only been observed in S2 cell culture under specific starving condition (Zacharogianni et al., 2014), we question the relevance of these finding to the study in vivo or in other organisms.
>> At this point, we kindly indicate to the reviewer that we have a manuscript in preparation regarding Sec body formation in mammalian cells. This manuscript is the direct consequence of this present study. The fact that Sec body form in mammalian cells that involved similar pathways is a clear sign of the relevance of these studies.

Rev2.11: The fact that Sec16 and Sec24 have IDRs and capability of phase separating into droplets is interesting and suggests a phase separation-dependent regulation on the secretory pathway. The work, however, did not address how these pathways affect phase separation of Sec16/24 and how secretory pathway is affected.
>>This is correct. This work does not address this. It is a whole new study that will take a long time to achieve. However, we agree that this aspect is critical and important, we believe that this is outside the scope of this study.

Editor point: "The only thing I might add for you to consider would relate to the ADP/ATP ratio. This clearly has major implications for AMPK activation".
To conclude on the AMPK involvement, we have used different ratio of ADP/ATP in our semi-intact cell system (presented in the **original Figure 7**). In the original experiment, we had used 0.5mM ATP and shown that it was enough to prevent Sec body formation. Here, we used different ratio of ADP/ATP as follows
-0.5mM ATP
-0.5mM ATP / 0.2mM ADP
-0.5mM ATP / 0.5mM ADP
-0.5mM ATP / 1.0mM ADP
However, addition of none of these ADP concentrations lifted the inhibition of Sec body formation by ATP.
Addition of ATP might inhibit AMKP whereas a higher ADP to ATP ratio would activate it, but this does not have an incidence on Sec body formation.
We conclude that AMPK would likely not be involved in Sec body formation.

References

Aguilera-Gomez, A., M.M. van Oorschot, T. Veenendaal, and C. Rabouille. 2016. In vivo vizualisation of mono-ADP-ribosylation by dPARP16 upon amino-acid starvation. *eLife*. 5.
Chang, T.-K., D.A. Lawrence, M.M. Lu, J. Tan, J.M. Harnoss, S.A. Marsters, P. Liu, W. Sandoval, S.E. Martin, and A. Ashkenazi. 2018. Coordination between Two Branches of the Unfolded Protein Response Determines Apoptotic Cell Fate. *Molecular cell*. 71:629-636.e625.
Gaddam, D., N. Stevens, and J. Hollien. 2012. Comparison of mRNA localization and regulation during endoplasmic reticulum stress in Drosophila cells. *Molecular Biology of the Cell*. 24:14-20.
Hollien, J., and J.S. Weissman. 2006. Decay of endoplasmic reticulum-localized mRNAs during the unfolded protein response. *Science*. 313:104-107.
Moore, K.A., and J. Hollien. 2012. The unfolded protein response in secretory cell function. *Annual review of genetics*. 46:165-183.
Nie, J., A. Liu, Q. Tan, K. Zhao, K. Hu, Y. Li, B. Yan, and L. Zhou. 2017. AICAR activates ER stress-dependent apoptosis in gallbladder cancer cells. *Biochemical and biophysical research*

© 2021. Published by The Company of Biologists under the terms of the Creative Commons Attribution License (https://creativecommons.org/licenses/by/4.0/).

communications. 482:246-252.
Tang, Y.C., B.R. Williams, J.J. Siegel, and A. Amon. 2011. Identification of aneuploidy-selective antiproliferation compounds. *Cell*. 144:499-512.
Yan, C., J. Liu, J. Gao, Y. Sun, L. Zhang, H. Song, L. Xue, L. Zhan, G. Gao, Z. Ke, Y. Liu, and J. Liu. 2019.
IRE1 promotes neurodegeneration through autophagy-dependent neuron death in the Drosophila model of Parkinson's disease. *Cell Death Dis*. 10:800.
Zacharogianni, M., A. Aguilera-Gomez, T. Veenendaal, J. Smout, and C. Rabouille. 2014. A stress assembly that confers cell viability by preserving ERES components during amino-acid starvation. *eLife*. 3.

**RIDD target RNAs** (from Hollien and Weissman, 2006, Moore and Hollien, 2015; Gaddam and Hollien (validated ones)	**Fold change in RNA expression between SCH and KRB** (LogFC)	**P-value**	**Protein function**
Indy	-1.10	E-08	Plasma membrane **transporter** of Krebs cycle intermediates
Miple 1 (midkine and pleiotrophin)	-1.03	0.017	Small **secreted** that binds heparin protein
CG9917	-0.961 (-0,24)	E-06	ND
PIG-Wa*	-1.535	E-04	**Phosphatidylinositol glycan anchor biosynthesis class Wa**
PIG-Z	-1.01	E-3	**class Z**
PIG-G	-0.928	E-05	**class G**
PIG-H	-0.675	E-3	**class H**
PIG-N	-0.621	0.03	**class N**
PIG-O	-0.574	E-3	**class O**
PIG-F	-0.563	0.05	**class F**
PIG-V	-0.532	0.016	**class V**
			**class T**
CG3984	-0.837	0.06	
Hpr1	-0.410	0.05	mRNA export from nucleus in response to heat stress
Blos 1 (mamm)<br>Blos 2	-0.483<br>-1.3	0.12	
Sema1b	-0.346	0.09	**Transmembrane protein** of the semaphorin protein [illegible]
Sema2a	-0.286	0.13	
Fatp	-0.187	0.3	**Transmembrane protein**
smt3 (sumo)	-0.175	0.66	The only Drosophila
CG6805	-0.141	0.5	**Inositol phosphate**
Jagn (jagunal)	-0.101	0.6	Conserved ER protein
CG6650			
(26-29 protease			
LanB1			
CG6805			

© 2021. Published by The Company of Biologists under the terms of the Creative Commons Attribution License (https://creativecommons.org/licenses/by/4.0/).

Second decision letter

MS ID#: JOCES/2021/258685

MS TITLE: Activation of salt Inducible Kinases, IRE1 and PERK leads to Sec bodies formation in Drosophila S2 cells

AUTHORS: Chujun Zhang, Wessel van Leeuwen, Marloes Blotenburg, Angelica Aguilera-Gomez, Sem Brussee, Rianne Grond, Harm Kampinga, and Catherine Rabouille

ARTICLE TYPE: Research Article

Thank you for sending your manuscript to Journal of Cell Science through Review Commons.

Thank you very much for the careful and extensive revisions to you manuscript. I am happy to tell you that your manuscript has been accepted for publication in Journal of Cell Science, pending standard ethics checks.

There are no additional reviewers' reports for this current version as I did not consider it necessary to return this to the reviewers.

© 2021. Published by The Company of Biologists under the terms of the Creative Commons Attribution License (https://creativecommons.org/licenses/by/4.0/).
